# Systematic literature review of immunoglobulin trends for anti-CD20 monoclonal antibodies in multiple sclerosis

**DOI:** 10.1007/s10072-022-06582-y

**Published:** 2023-01-17

**Authors:** Shiv Saidha, Judith Bell, Sydney Harold, Jose Marcano Belisario, Emma Hawe, Qiujun Shao, Kerri Wyse, Eric M. Maiese

**Affiliations:** 1 Division of Neuroimmunology and Neurological Infections, Johns Hopkins University School of Medicine, Baltimore, MD USA; 2 RTI Health Solutions, Manchester, UK; 3 Novartis Pharmaceuticals Corporation, East Hanover, NJ USA

**Keywords:** Multiple Sclerosis, Anti-CD20 monoclonal antibodies, Systematic literature review, Immunoglobulin, Infection

## Abstract

**Objective:**

To exp lore changes in immunoglobulin (Ig) levels for people with relapsing-multiple sclerosis (RMS) treated with ocrelizumab or ofatumumab and the relationship between Ig levels and infections.

**Methods:**

A systematic literature review (SLR) was conducted to identify clinical trials and real-world evidence (RWE) studies on Ig levels over time and studies on associations with infections for ocrelizumab and ofatumumab for people with RMS through 10 September 2021. Searches were conducted in Embase, MEDLINE, Cochrane Library, trial registries, and recent conference abstracts.

**Results:**

Of 1,580 articles identified, 30 reporting on 11 trials and 5 RWE studies were included. Ocrelizumab trials (*n* = 4) had 24–336 weeks of follow-up and reported decreasing Ig G (IgG) levels, while RWE (*n* = 5) had 52–78 weeks of follow-up and reported IgG to be stable or decrease only slightly. IgG levels were stable in ofatumumab trials (*n* = 5; 104–168 weeks of follow-up), but no RWE or longer-term studies were identified. No apparent association between decreased Ig levels and infections was observed during ofatumumab treatment (ASCLEPIOS I/II), while for ocrelizumab, the only data on apparent associations between decreased IgG levels and serious infection rates were for a pooled population of people with RMS or primary progressive MS.

**Conclusion:**

Decreasing IgG levels have been correlated with increased infection risk over time. IgG levels appeared to decrease over time in ocrelizumab trials but remained relatively stable over time in ofatumumab trials. Additional research is needed to understand differences between ocrelizumab and ofatumumab and identify people at risk of decreasing IgG levels and infection.

**Supplementary Information:**

The online version contains supplementary material available at 10.1007/s10072-022-06582-y.

## Introduction

Multiple sclerosis (MS) is a chronic autoimmune disease characterized by transient alterations in the blood–brain barrier, inflammation, demyelination, and neurodegeneration [1]. Relapsing MS (RMS) is the most common subtype of MS, typically beginning with a relapsing and remitting course that, after several years, in a subset of people, may transition to a clinical phenotype that is instead characterized by gradual neurological decline. This is referred to as secondary progressive MS (SPMS). On the other hand, approximately 10% of people with MS (PwMS) experience progressive accumulation of neurologic disability from the outset, termed primary progressive MS (PPMS) [1, 2]. Although it is common to differentiate MS subtypes according to these clinical phenotypes, rather than being clearly differentiated, these subtypes may instead form a continuum, representing different phases or stages of the same MS disease process as it evolves. Earlier in the MS disease course, adaptive over innate immune system–mediated inflammation is thought to predominate. Although innate immune system dysfunction may increase over time and potentially predominate in progressive MS, in later disease, adaptive immune dysfunction remains relevant [3, 4]. For example, meningeal B cell follicles are more common in progressive MS than relapsing–remitting multiple sclerosis (RRMS), and the same antigen-experienced B cell clones have been shown to present in the brain parenchyma, as in the meningeal follicles of PwMS [3, 5].

In people with RMS, treatment with the anti-CD20 monoclonal antibodies ofatumumab and ocrelizumab, both approved by the US Food and Drug Administration (FDA) and the European Medicines Agency (EMA), has been shown to delay disease progression, reduce relapses, and reduce new gadolinium enhancing and/or T2 lesion formation on magnetic resonance imaging brain scans [1, 6]. While the precise mechanism by which ofatumumab and ocrelizumab exert their therapeutic effects in MS is unknown, both are cytolytic monoclonal antibodies presumed to involve binding to CD20, a cell surface antigen present on pre-B and mature B lymphocytes. Following cell surface binding to B lymphocytes, ofatumumab and ocrelizumab result in antibody-dependent cellular cytolysis and complement-mediated lysis. The resulting immunosuppression may lead to an increased risk of serious infections. For example, among PwMS in the Swedish MS registry, treatment with the related anti-CD20 monoclonal antibody rituximab was associated with approximately 3 times greater odds of hospitalization for infection with COVID-19 relative to other disease-modifying therapies combined [7]. Ofatumumab may have a number of theoretical advantages with regard to immunosuppression, including administration via subcutaneous injection versus intravenous infusion and at a lower dose than ocrelizumab, resulting in a greater lymphatic compartment effect and overall less potential for deeper B cell depletion, as well as faster B cell repletion after discontinuation, and limited recovery of circulating B cells [8–10].

Some evidence from clinical trials and observational studies has suggested an association between Ig antibody levels and infection rates, as well as infection severity in PwMS. In particular, an association between reduced serum immunoglobulin G (IgG) and increased infection risk has been suggested [11]. Having an improved understanding of such risks is particularly relevant within the context of B cell–depleting therapeutic strategies because one of the main functions of B cells is antibody production. In a broader sense, people with RMS taking disease-modifying therapies (DMTs) that may interfere with the generation and/or release of Igs in response to infectious exposures may accordingly have a greater risk for serious infections—a particularly important therapeutic consideration during the COVID-19 pandemic. On the contrary, and somewhat unsurprisingly, because IgA plays an important role in adaptive immune protection at mucosal surfaces, it has been suggested that higher levels of serum IgA may be related to decreased infection risk [12]. Multiple clinical trials and real-world studies have reported Ig levels over time for patients with MS treated with ofatumumab and ocrelizumab. For example, the phase 3 ASCLEPIOS I/II study, with safety data up to 4 years, showed that mean IgG levels remained similar to baseline values for patients who used ofatumumab, and no associated increased risk of serious infections was reported [13].

In this systematic literature review (SLR), our principal objective was to review published data from randomized controlled trials (RCTs) and real-world evidence (RWE) studies on Ig levels over time between people with RMS treated with the currently approved anti-CD20 monoclonal antibodies, ofatumumab and ocrelizumab. Moreover, we also sought to determine if the incidence and severity of infectious disease adverse events correlate with Ig levels in people with RMS being treated with either ocrelizumab or ofatumumab.

## Methods

The target population for this SLR was people with RMS (including those with RRMS or with active SPMS) who were treated with either ocrelizumab or ofatumumab in either a clinical trial or an observational study setting.

The outcomes of interest from the included studies, where available, were as follows: mean or median IgM, IgG, and IgA levels at baseline and follow-up timepoints while on treatment with ocrelizumab or ofatumumab and their comparator DMTs, if applicable; changes in mean or median IgM, IgG, and IgA from baseline to different timepoints; and the percentage of the study population below a given threshold for IgM, IgG, and IgA at baseline and at different timepoints while on treatment.

Methods of this SLR were consistent with those outlined in the Cochrane Handbook for Systematic Reviews of Interventions [14]. Following a study protocol with prespecified search terms, experienced research librarians conducted electronic searches to identify English-language publications with publication dates from the initiation of the databases searched until 10 September 2021. We placed no limitation on geography and searched the following databases: MEDLINE and MEDLINE In-Process (using the PubMed platform); Embase (using the Elsevier Platform); and the Cochrane Library, including the Cochrane Central Register of Controlled Trials (CENTRAL) and the Cochrane Database of Systematic Reviews. In addition, selected conference proceedings, trial registries, and regulatory websites were searched. To minimize the risk of missing eligible studies, we also performed a manual search by screening the bibliographies of identified SLRs and meta-analyses and included studies.

**Fig. 1:**
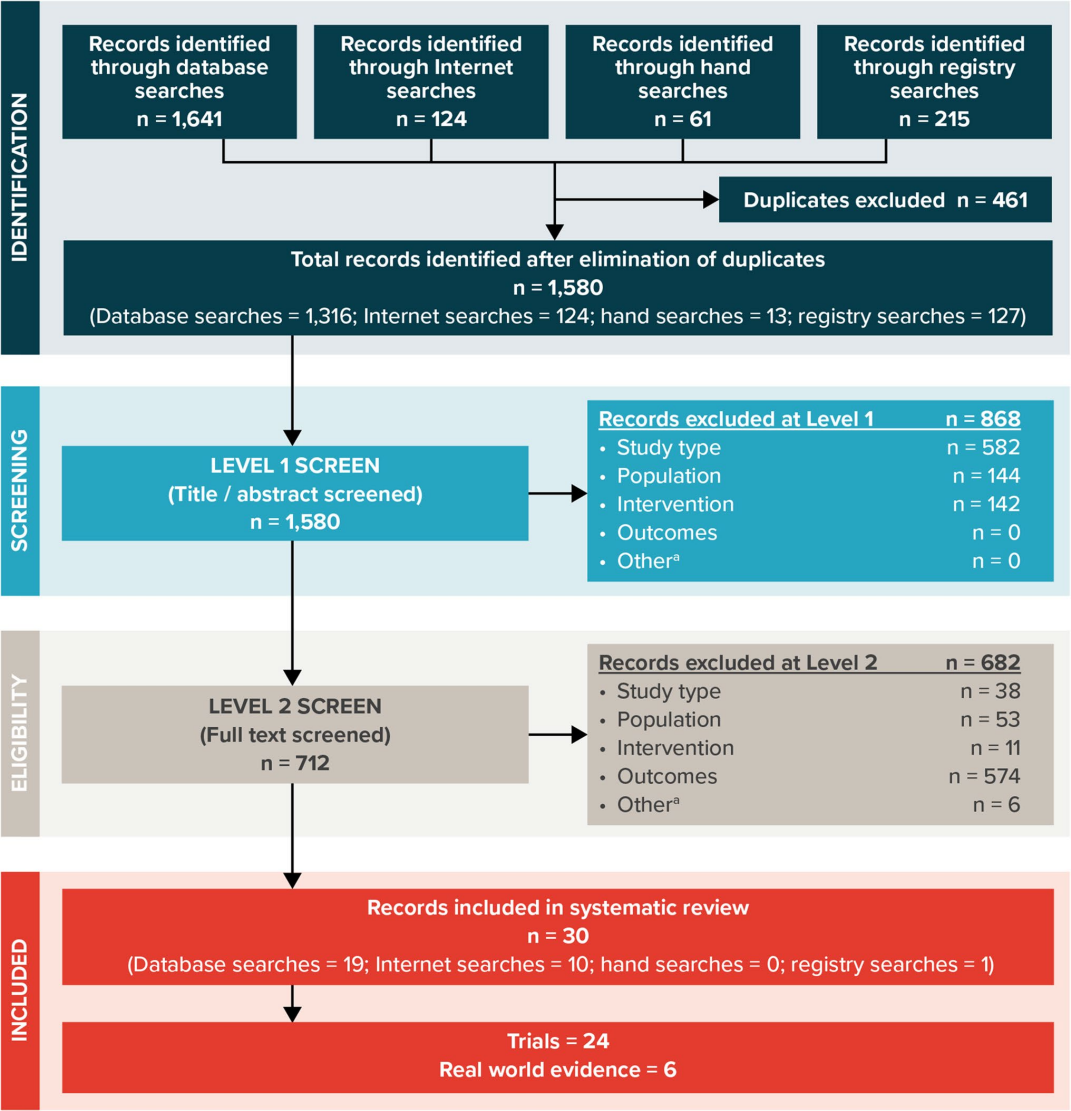
PRISMA diagram for the systematic literature review. PRISMA = Preferred Reporting Items for Systematic Reviews and Meta-Analyses [15]. ^a^The category “Other” includes duplicate references and conference abstracts before 2017

Screenings of publications for inclusion were based on prespecified inclusion and exclusion criteria (Table 1). Publications were screened by 1 researcher with a 10% random check conducted by a second researcher. The inclusion and exclusion process was documented using a Preferred Reporting Items for Systematic Reviews and Meta-Analyses (PRISMA) diagram (see Fig. 1).

**Table 1:** List of criteria for the inclusion and exclusion of studies at level 1 (title and abstract) screening and level 2 (full-text) screening

Criterion	Included	Excluded
Population	Level 1 and level 2 inclusion criteria: ▪ Adults with RMS, including RRMS and active SPMS ▪ Publications with a mixed population that include separate data for people with RMS were included	Level 1 and level 2 exclusion criteria: ▪ Other forms of MS ▪ Pediatric cases
Interventions and comparators	Level 1 and level 2 inclusion criteria: ▪ To be included in the review, a study must have had at least 1 of the interventions of interest in at least 1 study arm: - Ocrelizumab - Ofatumumab	Level 1 and level 2 exclusion criteria: ▪ Studies that do not include at least 1 intervention of interest
Study design	Level 1 and level 2 inclusion criteria: ▪ Phase 2–4 randomized, controlled, prospective, clinical trials ▪ Single-arm, prospective clinical trials ▪ Long-term follow-up studies of prospective clinical trials ▪ Real-world evidence (including observational studies, cohort studies, case–control studies, cross-sectional studies, registry studies, and retrospective studies) ▪ Post hoc and pooled analyses of trials or real-world evidence ▪ Meta-analyses ▪ Systematic reviews (including meta-analyses)^ a^	Level 1 exclusion criteria: ▪ Epidemiological and/or ecological studies^ b^ ▪ Phase 1 trials ▪ Case reports ▪ Commentaries, letters, or editorials (publication type) ▪ Consensus reports ▪ Nonsystematic reviews ▪ Preclinical studies ▪ Surveys ▪ Questionnaires ▪ Animal studies (not in humans) ▪ Studies pooling MS types (other than RRMS and active SPMS) and not presenting results separately Level 2 exclusion criteria: ▪ Systematic reviews^a^
Outcomes^c^	Level 2 inclusion criteria: ▪ To be included in the review, a study must report at least 1 of the outcomes of interest: - Ig levels (IgM, IgG, IgA) over time ^d^ - Relationship between Ig levels and infections	Level 2 exclusion criteria: ▪ Studies that do not report at least 1 of the outcomes of interest
Language	Level 1 and level 2 inclusion criteria: ▪ English language	Level 1 and level 2 exclusion criteria: ▪ Non-English language
Date	Level 1 and level 2 inclusion criteria: ▪ No date limit for full-text publications ▪ 1 January 2017 to the present for conference abstracts	Level 1 and level 2 exclusion criteria: ▪ Conference abstracts published before 2017

*Ig*, immunoglobulin; *MS*, multiple sclerosis; *RMS*, relapsing multiple sclerosis; *RRMS*, relapsing–remitting multiple sclerosis; *SPMS*, secondary progressive multiple sclerosis

If it was unclear whether a study met any criterion during the level 1 screening process, the study was progressed to full-text screening to confirm its inclusion in the review

^a^Systematic reviews were included at level 1 screening, hand searched for identification of primary studies, and then excluded at level 2 screening

^b^Studies on the incidence and prevalence of the disease that do not provide further outcomes of interest

^c^Screening of studies for relevant outcomes was conducted only at level 2 (full-text) screening

^d^This may relate to studies with baseline and 1 follow-up measure, or multiple measures during the study follow-up

One reviewer abstracted data from included studies into detailed evidence tables (see Appendix A, Supplemental Material); a second reviewer checked all abstractions against the original source. Data in the evidence tables included information on study authors, year, country, and funding source; RMS population(s) studied; trial design or data source used; and other study characteristics. The evidence tables also included key study endpoints. Where key information was available within figures only, we digitized these data using DigitizeIt software (DigitizeIt; I. Bormann; Braunschweig, Germany), and the digitized data were checked by a second researcher. Owing to heterogeneity in the studies and reporting of outcomes, and the lack of common comparators across the studies, indirect comparison of aggregate data was not considered to be feasible. Therefore, no statistical analysis was conducted; here, we present a summary of the data reported in the identified studies separately.

## Results

### SLR results

The electronic database, internet searches, hand searches, and registry searches were conducted 10–16 September 2021 and yielded a total of 1,580 records (titles and abstracts) for manual level 1 screening of titles and abstracts (databases = 1,316; internet searches = 124; hand searches = 13; registry searches = 127). After the initial (level 1) screening of titles and abstracts according to the inclusion and exclusion criteria in Table 1, 712 publications (database searches = 492; internet searches = 106; hand searches = 6; registry searches = 108) were progressed to level 2 full-text screening. At the level 2 screening, 30 publications (database searches = 19; internet searches = 10; hand searches = 0; registry searches = 1) met the predefined inclusion criteria and thus were selected for data extraction. Figure 1 depicts the volume of publications included and excluded at each stage of screening in a PRISMA flow diagram.

### Study characteristics

#### Clinical trials

The 24 included trial publications reported data on 11 trials (ASCLEPIOS I and II, APLIOS, APOLITOS, ALITHIOS, OBOE, OPERA I and II, OMS115102, VELOCE, and NCT00676715) (Table 2). Of these, OPERA I and II were reported only as pooled data. APLIOS, APOLITOS, and ALITHIOS were reported only as pooled data with ASCLEPIOS I and II, whereas ASCLEPIOS I and II each were reported separately and in addition were pooled. Therefore, results from 9 trial populations were included.

**Table 2:** Study design and outcomes reported

Trial name/author, country	Study design and duration	Treatment (n)	Outcomes reported								
			Mean or median			Change from baseline			% achieving a certain level		
			IgA	IgG	IgM	IgA	IgG	IgM	IgA	IgG	IgM
Clinical trials											
ASCLEPIOS I [16], multinational NCT02792218	Phase 3 RCT, multicenter, double-blind, 120 weeks	Ofatumumab (*n =* 465)		✓	✓						
		Teriflunomide (*n =* 462)									
ASCLEPIOS II [16], multinational NCT02792231	Phase 3 RCT, multicenter, double-blind, 120 weeks	Ofatumumab (*n =* 481)		✓	✓						
		Teriflunomide (*n =* 474)									
ASCLEPIOS I and II pooled	As above	As above		✓	✓		✓ ^a^	✓ ^a^		✓	✓
ASCLEPIOS I/II, APLIOS, APOLITOS, ALITHIOS [17], NCT02792218, NCT02792231, NR, NCT03560739, NCT03650114	ASCLEPIOS I/II (phase 3 RCT), APOLITOS (phase 2 RCT), APLIOS (phase 2 RCT), (long term) ALITHIOS (ongoing phase 3, open-label, single-arm, 5-year extension study) Results reported up to 3.5 years	ASCLEPIOS I/II: ofatumumab vs. teriflunomide		✓	✓		✓ ^a^	✓ ^b^		✓	✓
		APOLITOS: ofatumumab 20 mg vs. placebo									
		APLIOS: ofatumumab with pre-filled syringe vs. ofatumumab with auto-injector									
		ALITHIOS: ofatumumab									
OBOE 18], multinational NCT02688985	Phase 4, open-label, RCT 52 weeks	Ocrelizumab 600 mg (*n =* 79 of 100 total people with RMS with available CSF samples)		✓^c^	✓^c^		✓ ^a,c^	✓ ^a,c^			
VELOCE [19], US and Canada NCT02545868	Phase 3, open-label, multicenter RCT 24 weeks	Ocrelizumab 600 mg (*n =* 68)		✓	✓						
		Control (*n =* 34)									
OPERA I and II pooled [20], multinational NCT01247324 and NCT01412333	Phase 3, multicenter, double-blind RCT 96 weeks with 3-year open-label extension	Ocrelizumab (*n =* 410) (*n =* 417)	✓	✓	✓	✓ ^a^	✓ ^b^	✓ ^b^	✓	✓	✓
		Interferon beta-1a (*n =* 411) (*n =* 418)									
OMS115102 [21], multinational NCT00640328	Phase 2, multicenter, double-blind, crossover, dose-finding RCT 48-week treatment period followed by an individualized treatment period of up to 2 years	Ofatumumab 100 mg to Week 24, then placebo (*n =* 8)				✓	✓	✓			
		Ofatumumab 300 mg to Week 24, then placebo (*n =* 11)									
		Ofatumumab 700 mg to Week 24, then placebo (*n =* 7)									
		Placebo to Week 24, then ofatumumab 100 mg (*n =* 4)									
		Placebo to Week 24, then ofatumumab 300 mg (*n =* 4)									
		Placebo to Week 24, then ofatumumab 700 mg (*n =* 4)									
NCT00676715 [22], multinational	Phase 2, multicenter, double-blind RCT 72-week treatment period followed by a 78-week treatment-free period	Placebo (*n =* 54)					✓	✓			
		Ocrelizumab 600 mg (*n =* 55)									
		Ocrelizumab 2,000 mg (*n =* 55)									
		Interferon beta-1a (*n =* 54)									
Real-world evidence studies											
Prezioso et al. [23], Italy	Single-arm interventional study 52 weeks	Ocrelizumab (*n =* 42)		✓	✓						
van Lierop et al. [24], Netherlands	Observational cohort study Median follow-up, 91 weeks	Ocrelizumab direct switch (*n =* 27)		✓							
		Ocrelizumab indirect switch (*n =* 15)									
Edgar et al. [25], US	Retrospective chart review Duration of follow-up NR	Ocrelizumab super response ^d^ (*n =* 13)		✓	✓						
		Ocrelizumab remaining population (*n =* 122)									
Evertsson et al. [26], Evertsson et al. [27], US	Retrospective cohort study 52 weeks	Ocrelizumab (*n =* 161)		✓	✓		✓	✓			
Lopez Ruiz et al. [28], Spain	Retrospective observational study Mean follow-up, 82 weeks	Ocrelizumab (*n =* 52)		✓	✓					✓	✓

*CSF*, cerebrospinal fluid; *IgG*, immunoglobulin G; *IgM*, immunoglobulin M; *NR*, not reported; *RCT*, randomized controlled trial; *RMS*, relapsing multiple sclerosis

^a^Reports percentage change from baseline only

^b^Reports both absolute change from baseline and percentage change from baseline data

^c^CSF immunoglobulin levels, not serum

^d^Rapid improvement in symptom profiles, relapse free, and with magnetic resonance imaging stability

Of the 11 included trials, ASCLEPIOS I and II, APLIOS, ALITHIOS, OBOE, OPERA I and II, OMS115102, and NCT00676715 were multinational; APOLITOS was conducted in Japan and Russia; and VELOCE was conducted in the United States (US) and Canada. Ocrelizumab was studied in 5 trials (OBOE, OPERA I and II, VELOCE, and NCT00676715), and ofatumumab in 6 trials (OMS115102, ASCLEPIOS I and II, APLIOS, APOLITOS, and ALITHIOS). ASCLEPIOS I and II [16] were phase 3, multicenter, double-blind, RCTs sponsored by Novartis Pharmaceuticals. The trials included people with RMS who received treatment with either ofatumumab (*n* = 946) or teriflunomide (*n* = 936) for up to 130 weeks. Wiendl et al. [17] further pooled ASCLEPIOS I and II data with the phase 2 RCT APOLITOS, phase 2 RCT APLIOS, and the ongoing, phase 3, open-label, 5-year extension ALITHIOS trial (with 3.5 years of data available at the time of this SLR), all of which included people with RMS treated with ofatumumab throughout and were sponsored by Novartis Pharmaceuticals. VELOCE [19] and OPERA I and II [20] were also phase 3 RCTs, both of which were sponsored by Hoffmann-La Roche. VELOCE [19] was a 24-week, phase 3, open-label, multicenter RCT that included assessment of the effect of ocrelizumab treatment on response to vaccines. This trial included adults with RRMS who were randomized either to ocrelizumab (*n* = 68) or to a control group (*n* = 34) in which people either continued their current interferon beta therapy or received no DMT. OPERA I and II [20] were phase 3 multicenter, double-blind RCTs that included people with RMS who were randomly assigned to receive ocrelizumab at a dose of 600 mg by means of intravenous infusion every 24 weeks (*n* = 410, OPERA I; *n* = 417, OPERA II) or interferon beta-1a (*n* = 411, OPERA I; *n* = 418, OPERA II) at a dose of 44 μg administered subcutaneously 3 times weekly throughout the 96-week treatment period.

OMS115102 [21] was a phase 2 multicenter, double-blind, crossover, dose-finding RCT that was sponsored by GlaxoSmithKline. This trial included people with RRMS who received treatment with either ofatumumab or placebo across 6 experimental cohorts receiving varying doses of intravenous ofatumumab (100 mg, 300 mg, or 700 mg; *n* = 26) crossing over to placebo, or placebo crossing over to intravenous ofatumumab (*n* = 12) during a 48-week treatment period. NCT00676715 [22] was also a phase 2 RCT with a parallel-group, double-blind design sponsored by Genentech, Inc. This trial included people with RRMS who were randomized to either 2 placebo intravenous infusions at 15-day intervals (*n* = 54), 2 infusions of 300-mg ocrelizumab at 15-day intervals with infusion reaction prophylaxis (*n* = 55), or open-label 30-μg interferon (IFN) β administered intramuscularly once a week (*n* = 54) over a 72-week treatment period.

OBOE [18] was the only phase 4 open-label RCT trial identified in this SLR. This trial was also sponsored by Genentech, Inc., and included a population of 79 of 100 total people with RMS with available cerebrospinal fluid (CSF) samples who were treated with ocrelizumab and had undergone lumbar punctures. Ocrelizumab was administered as two 300-mg intravenous (IV) infusions on Days 1 and 15, then as a single infusion of 600 mg on weeks 24 and 48. Participants received a lumbar puncture before the start of dosing with ocrelizumab and a second lumbar puncture at week 12.

Among the 11 included trials, 5 trials required a diagnosis of RMS in accordance with the 2010 revised McDonald criteria, 5 trials specified a participant age range between 18 and 55 years, and all required an Expanded Disability Status Scale (EDSS) score, which ranged between 0 and 6 across trials. Detailed tables presenting the study design for each trial, including eligibility criteria (Table S1) and baseline characteristics (Table S2), are included in Appendix A (Supplemental Material).

#### Real-world studies

Six RWE publications reported data on 5 studies (Table 2), all of which studied ocrelizumab. Of the included studies, 2 were conducted in the US, 1 in the Netherlands, 1 in Italy, and 1 in Spain. Three studies were retrospective and 2 were prospective. The studies had small sample sizes ranging from 42 to 161 participants, with follow-up times ranging from 52 to 78 weeks; 3 of the 6 included publications were conference abstracts only. Detailed tables presenting the study design, including eligibility criteria (Table S3) and baseline characteristics (Table S4), for the RWE studies are included in Appendix A (Supplemental Material).

### Key outcomes

Among the clinical trial studies included in the review, for both IgG and IgM levels, mean or median values were reported in 7 of the 9 included trial populations, change from baseline in 6, and percentage of participants achieving a certain level in 3 trial populations (Table 2). IgA data were reported the least across the trials, with mean or median values for IgA reported in just 1 trial population, change from baseline in 2 trial populations, and percentage of people achieving a certain level in just 1 trial population. Of the 9 trial populations included in the SLR, only 3 reported data on the association of Ig levels with infection. Most data came from four large phase 3 RCTs: ASCLEPIOS I and II [16, 29–33] and OPERA I and II [20, 34–40]. NCT00676715 reported limited data [22]. While the VELOCE trial did report change in Ig levels, the trial focused on immune responses and the effectiveness of vaccinations in ocrelizumab-treated people over only 24 weeks [19]. OBOE only reported cerebrospinal fluid (CSF) Ig levels [18], while OMS115102 investigated much higher intravenous doses of ofatumumab than the marketed subcutaneous dose and included only 38 participants [21].

Among the real-world studies included in the review, mean or median IgG levels were reported for all 5 of the included studies, and IgG change from baseline and percentage of individuals achieving a certain level were reported in 1 study each (Table 2). Mean or median IgM levels were reported in 4 of the 5 RWE studies, with IgM change from baseline values reported in just 1 study. No data were provided for IgA levels in any of the included RWE studies.

### IgG

**Fig. 2:**
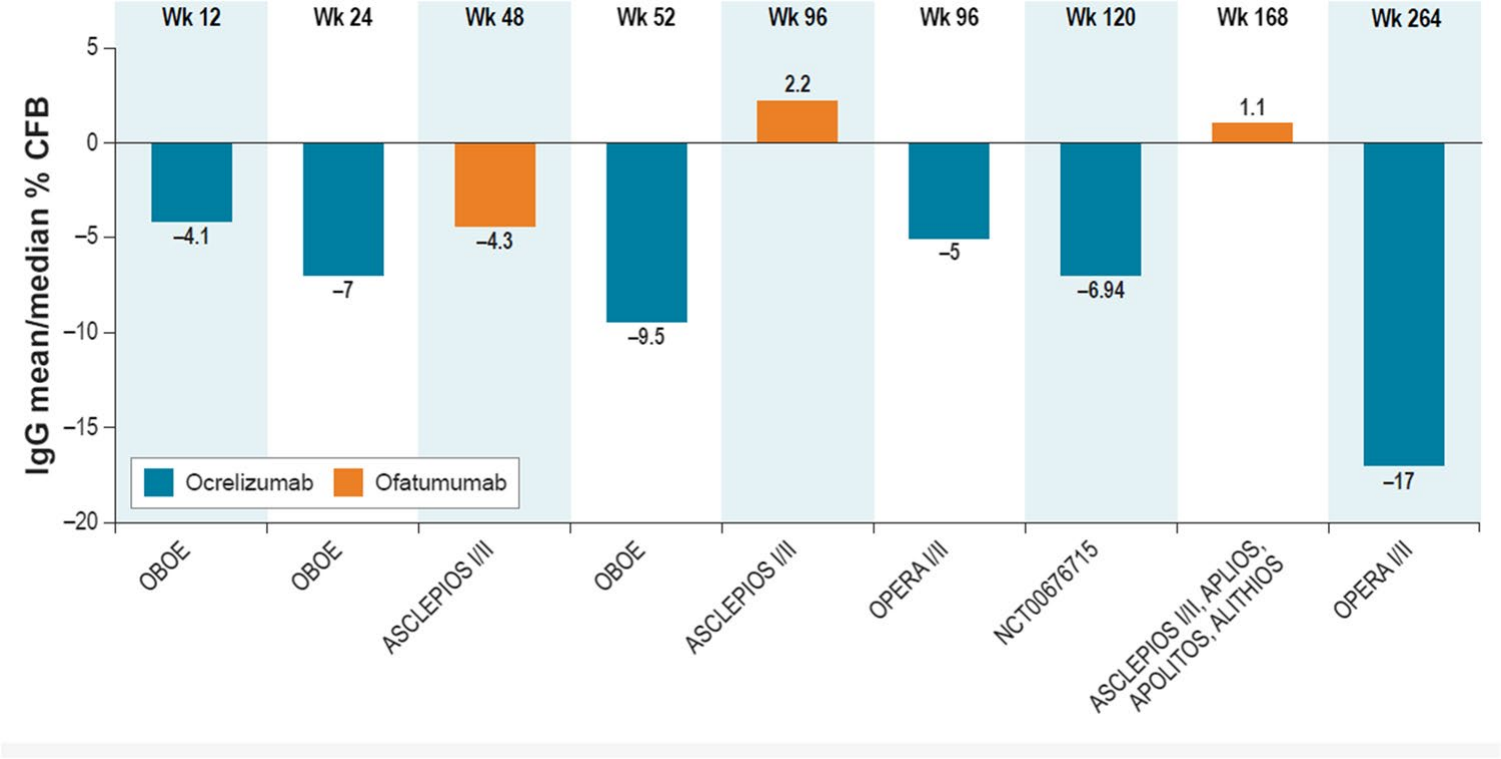
Mean/median percentage change from baseline in IgG levels: clinical trials of ocrelizumab and ofatumumab. CFB = change from baseline; CSF = cerebrospinal fluid; IgG = immunoglobulin G; LLN = lower limit of normal. Note: Changes in IgG levels are for summary purposes only; owing to heterogeneity in trial designs and outcomes, cross-trial comparisons should not be made. Detailed outcomes of clinical trials are presented in Appendix B, Supplemental Material. OBOE examined only CSF IgG levels and not serum IgG levels. ASCLEPIOS I/II: median IgG levels are presented; LLN for serum IgG defined as 5.65 g/L [17]. VELOCE: mean IgG levels are presented; LLN for serum IgG defined as 4.6 g/L [37]. OPERA I/II: mean IgG levels are presented; LLN for serum IgG defined as 5.65 g/L [35]. NCT00676715: mean IgG levels are presented. ASCLEPIOS I/II, APLIOS, APOLITOS, ALITHIOS pooled: mean IgG levels are presented; LLN for serum IgG defined as 7.0 g/L [29]. Treatment interruption due to notably low IgG levels (> 20% below LLN) and treatment discontinuation were reported for 0.1% and 0.2% of patients, respectively [17]

The most reported outcome was change in IgG levels (Fig. 2; Table S5, Appendix B). Four trial populations of ocrelizumab with 24 weeks to 336 weeks of follow-up reported a decrease in IgG levels over time. In 5 trial populations of ofatumumab with 104 to 168 weeks of follow-up, a transient decrease in IgG levels occurred at week 48, but decreases in IgG levels were not observed at later time points (Fig. 2).

In the OPERA I and II trials, a decrease in mean IgG levels was observed for the ocrelizumab treatment arm over 336 weeks [38]. These trials reported a mean change of –5% at week 96 and of –17% at week 264 in the ocrelizumab arm (Fig. 2) [36, 38]. In the IFN β-1a arm, after a mean increase up to week 96, a decrease was subsequently observed after the switch to ocrelizumab treatment (Table S5) [38]. Over a period of up to 7 years, IgG levels decreased at an average rate of 0.33 g/L per year (− 3% per year). At the latest recorded timepoint of week 312, 7.7% of people treated with ocrelizumab throughout had an IgG level less than the lower level of normal (LLN). NCT00676715 also reported a mean reduction (–6.94% vs baseline) in IgG level at week 120 in people who received 4 cycles of ocrelizumab [22] (Fig. 2). In the VELOCE study, mean IgG levels were 10.25 g/L at baseline, 10.36 g/L at week 12, and 10.21 g/L at week 24, although it should be noted that this vaccine response study had a much shorter duration of only 24 weeks.

In the ASCLEPIOS I and II trials, a transient drop in median IgG levels was observed with ofatumumab, returning to baseline value by week 72. These trials reported a mean change of –4.3% at week 48 and of + 2.2% at week 96 [41] (Fig. 2). Mean IgG levels remained stable over up to 3.5 years of treatment and above the LLN of 5.65 g/L [17]. In the pooled ASCLEPIOS I/II, APOLITOS, APLIOS, and ALITHIOS population, after a transient drop through week 48 IgG levels returned to baseline levels, which were maintained across 3.5 years of ofatumumab treatment [17] (Fig. 2).

**Fig. 3:**
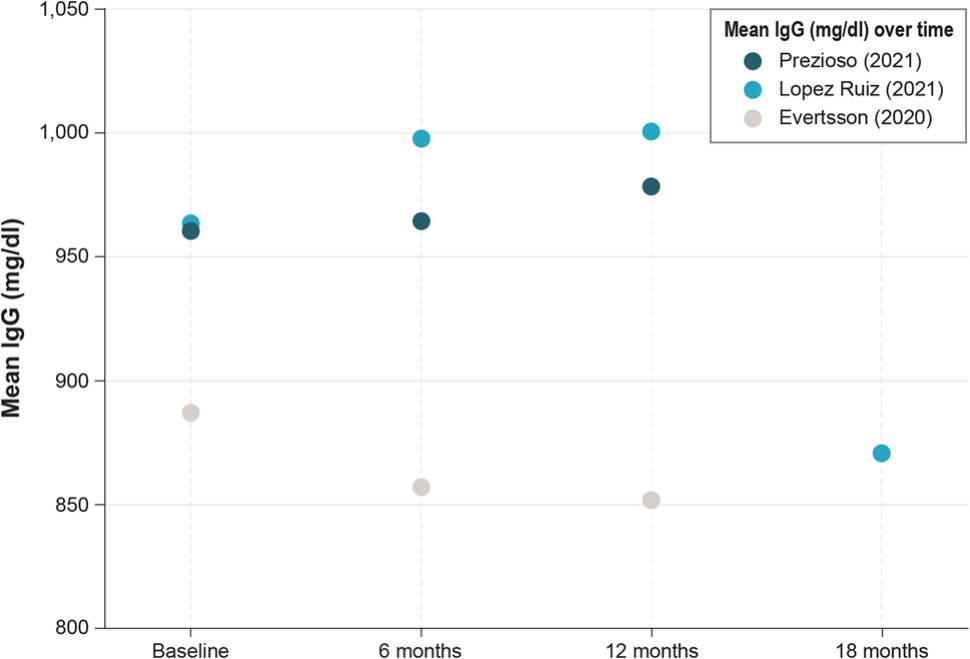
Mean IgG levels over time with ocrelizumab treatment: real-world studies. IgG = immunoglobulin G. Note: IgG levels over time are for summary purposes only; owing to heterogeneity in study designs and outcomes, cross-study comparisons should not be made. Detailed outcomes of real-world studies are presented in Appendix B, Supplemental Material

Real-world studies (all of which studied ocrelizumab) generally reported that IgG levels decreased slightly over time or remained stable, although results were variable and it was often unclear if any decrease was statistically significant (see Table S6, Appendix B). The RWE studies performed by Lopez Ruiz et al. [28], Evertsson et al. [26], and Edgar et al. [25] all reported similar patterns in which an overall decrease in median and mean IgG levels over time was observed with ocrelizumab treatment (Fig. 3 and Table S6). That being said, information on statistical significance was not reported by Edgar et al. [25] or Lopez Ruiz et al. [28]; in the study by Evertsson et al. [26], the change in IgG levels was significant in one analysis but not in another. More specifically, Evertsson et al. [26] reported a mean change of –0.16 g/L (95% confidence interval [CI], − 0.31 to − 0.01; *P* = 0.039) with each ocrelizumab infusion by mixed-effects modeling, but analysis by generalized estimating equations was not significant (*P* = 0.102). They further reported mean IgG levels by subgroups, with the largest decrease at 52 weeks found in people aged > 50 years. Lopez Ruiz et al. [28] reported that no participants exhibited IgG levels < LLN at 78 weeks. It should also be noted that Prezioso et al. [23] (in a single-arm interventional study) and van Lierop et al. [24] (in a cohort study of individuals switching from natalizumab to ocrelizumab either directly or indirectly because of progressive multifocal leukoencephalopathy risk) reported slight increases from baseline in mean and median IgG levels over time with ocrelizumab treatment. Prezioso et al. [23] reported that IgG levels had a stationary trend over time (*P* < 0.05) during the 12 months ocrelizumab treatment, whereas van Lierop et al. [24] did not comment on the results. However, both studies had sample sizes of only 42 participants (Fig. 3 and Table S6). Lopez Ruiz et al. [28] also reported an increase from baseline to week 52, before levels decreased.

### IgM

**Fig. 4:**
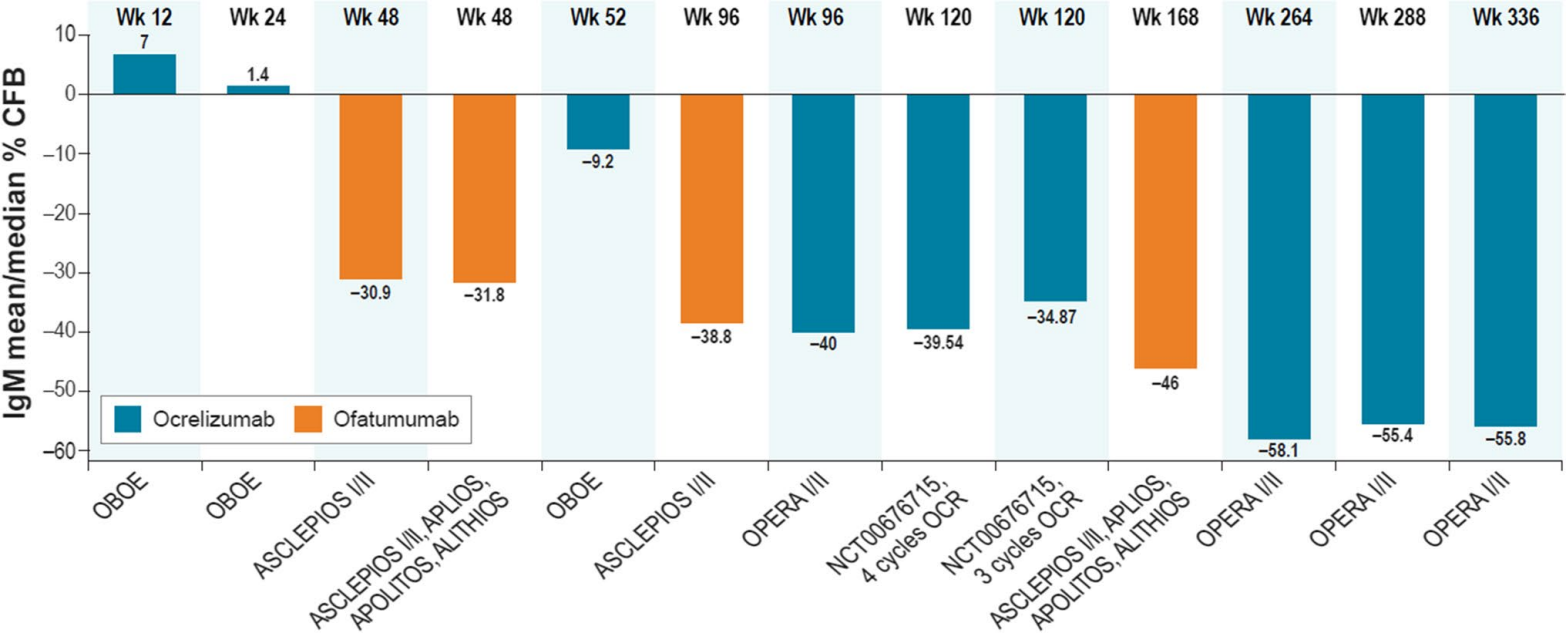
Mean/median percentage change from baseline in IgM levels: clinical trials of ocrelizumab and ofatumumab. CFB = change from baseline; CSF = cerebrospinal fluid; IgM = immunoglobulin M; LLN = lower limit of normal; OCR = ocrelizumab. Note: Changes in IgM levels are for summary purposes only; owing to heterogeneity in trial designs and outcomes, cross-trial comparisons should not be made. Detailed outcomes of clinical trials are presented in Appendix B, Supplemental Material. OBOE examined only CSF IgM levels and not serum IgM levels. ASCLEPIOS I/II: median IgM levels are presented; LLN for serum IgM defined as 0.4 g/L [17]. VELOCE: mean IgM levels are presented; LLN for serum IgM defined as 0.37 g/L [37]. OPERA I/II: mean IgM levels are presented; LLN for serum IgM defined as 0.4 g/L [35]. NCT00676715: mean IgM levels are presented. ASCLEPIOS I/II, APLIOS, APOLITOS, ALITHIOS pooled: mean IgM levels are presented; LLN for serum IgM defined as 0.4 g/L [29]. Treatment interruption due to notably low IgM levels (> 10% below LLN) and treatment discontinuation were reported for 9.1% and 3.3% of patients, respectively [17]

IgM was reported to decrease over time in both ocrelizumab and ofatumumab trials (Fig. 4, Table S6). In the same trials that reported IgG, IgM levels decreased over time for both ocrelizumab and ofatumumab.

The OPERA I and II trials reported a consistent decrease in IgM levels from baseline through week 336, with a mean relative reduction of 55.8% at week 336 for all ocrelizumab participants combined [38] (Table S5). VELOCE reported a decrease in mean IgM levels from baseline to week 24 in the ocrelizumab treatment group, while IgM levels remained stable in the control group [19]. NCT00676715 reported a mean reduction of 34.87% in IgM with 3 cycles of ocrelizumab and 39.54% with 4 cycles of ocrelizumab at week 120 [22].

The ASCLEPIOS I and II trials reported a mean decrease of 30.9% at week 48 and 38.8% at week 96 for people treated with ofatumumab [41] (Fig. 4). Similarly, for the pooled data from the ASCLEPIOS I and II trials with data from the APOLITOS, APLIOS, and ALITHIOS studies, mean decreases of 31.8% and 46% were observed at week 48 and week 168, respectively, for participants treated with ofatumumab [17] (Fig. 4).

The 4 RWE studies reporting on IgM values studied ocrelizumab, and all reported a decrease in IgM levels over time with ocrelizumab treatment (Table S6). Lopez Ruiz et al. [28] reported that 11 of the 52 included participants had IgM levels < LLN at 78 weeks.

### IgA

IgA data were reported for only 2 trial populations (Table S5). In the OPERA I and II trials, a decrease in mean IgA levels was observed for the ocrelizumab treatment arm over 336 weeks, and after a mean increase in the IFN β-1a arm up to week 96, a decrease was observed after the switch to ocrelizumab treatment [36, 39]. This trial also reported a change from baseline in IgA levels, with a mean 21.3% decline at week 264 reported for people treated with ocrelizumab. OPERA I/II was also the only trial to report the percentage of participants achieving a certain level for IgA. At baseline, 1.5% of participants had IgA levels < LLN in the ocrelizumab arm and 1.2% in the IFN β-1a–treated cohort. At the latest recorded timepoint of week 312, 7.5% of the ocrelizumab cohort and 3.9% of the IFN β-1a cohort had IgA levels < LLN [38]. IgA was not reported in clinical trials of ofatumumab but was reported in the OMS115102 dose-ranging study of high-dose intravenous ofatumumab [21]. In OMS115102, participants switching from placebo to ofatumumab 100 mg (considerably higher than the approved dose of 20 mg administered subcutaneously) had a mean change from baseline of − 0.07 (standard deviation [SD], 0.18) g/L at week 24 and − 0.00 (SD, 0.24) g/L at week 48. For those receiving placebo followed by ofatumumab 300 mg, mean change from baseline at week 24 was 0 (SD, 0.44) g/L, and 0.05 (SD, 0.21) g/L at week 48. Finally, for people receiving placebo then ofatumumab 700 mg, mean change from baseline was 0.49 (SD, 0.35) g/L at week 24 and 0.29 (SD, 0.26) g/L at week 48.

No data were provided on specific IgA levels in any of the included RWE studies. Evertsson et al. [26] reported that levels of IgA in blood were not affected by 52 weeks of ocrelizumab treatment based on the results of a retrospective study but presented no specific IgA values.

#### Association of Ig levels with infection

Three trial populations reported data on the association of Ig levels with infection: ASCLEPIOS I/II (trial population 1); ASCLEPIOS I/II, APLIOS, APOLITOS, ALITHIOS (trial population 2); and OPERA I/II, pooled with ORATORIO in most publications (trial population 3) (Table 3). Over all postbaseline visits, 14.2% of ASCLEPIOS I/II participants receiving ofatumumab had IgG below LLN. The proportion of ASCLEPIOS I/II participants on ofatumumab who experienced at least 1 infection within 1 month prior to and until 1 month after IgG below LLN was 27.6% (37 of 134; 3 serious) versus 50.6% (410 of 810) with IgG at or above LLN (21 serious) [29]. The proportion of participants on ofatumumab who experienced at least 1 infection within 1 month prior to and until 1 month after IgM below LLN was 31.1% (52 of 167; 2 serious), versus 51.5% (400 of 777) with IgM at or above LLN (18 serious) [29]. No association was observed with decreased IgM or IgG levels and increased risk of serious/nonserious infections in individuals treated with ofatumumab. Wiendl et al. [17] (ASCLEPIOS I/II, APLIOS, APOLITOS, and ALITHIOS trials) also reported on the association between IgG levels and infection risk and between IgM levels and infection risk for individuals undergoing long-term treatment with ofatumumab, concluding that no apparent association was observed between low IgG or IgM levels and risk of serious infections after 3.5 years of ofatumumab treatment. The OPERA I/II trials also reported data on the association of IgG, IgM, and IgA levels with infection. A numerical trend of lower rates of serious infections among higher quartiles of baseline IgG level was observed (by baseline IgG quartile, serious infections rates per 100 participant-years [95% CI] were as follows: Q1, 1.63 [0.95–2.61]; Q2, 1.55 [0.90–2.48]; Q3, 1.51 [0.86–2.45]; and Q4, 1.11 [0.57–1.94]) [37]. However, most data on association of Ig levels with infection rates reported for the OPERA I/II trials were pooled with the ORATORIO trial, which itself did not meet eligibility criteria for this SLR because it included people with PPMS. For IgG levels, 14 serious infections occurred during a drop in IgG level < LLN as compared with 208 serious infections for those during IgG levels ≥ LLN [35, 36]. The authors reported that an apparent association between decreased levels of IgG and rates of serious infections was observed, although the types, severity, duration, and outcomes of these infections were similar to those of the overall population treated with ocrelizumab and to the general MS population [35, 36]. For IgM levels, OPERA I/II reported that 71 serious infections occurred during a drop in IgM level < LLN as compared with 151 serious infections for those during IgM levels ≥ LLN [35, 36]. OPERA I/II were also the only trials to report data on the association of IgA levels with infection, with 7 serious infections occurring during a drop in IgA levels < LLN, compared with 215 when IgA levels were ≥ LLN [35].

**Table 3:** Association with infections, as reported in clinical trials

Trial name, country	IgA	IgG	IgM
ASCLEPIOS I/II trials: ASCLEPIOS I/II trials [29] [abstract], Multinational	NR	27.6% (37/134) reported infections during a drop in IgG < LLN (3 serious) vs. 50.6% (410/810) with IgG ⩾ LLN (21 serious) The most common infection was nasopharyngitis. Overall, 7/20 participants with concurrent IgG < LLN and lymphopenia and/or neutropenia reported infections; none were serious From presentation: 20/134 had concurrent IgG < LLN and lymphopenia and/or neutropenia. Of these 7 participants reported infection: upper respiratory tract infection = 2, nasopharyngitis = 2, UTI = 2, alveolar osteitis = 1 No association was observed with decreased Ig levels and increased risk of serious/nonserious infections or infections in conjunction with lymphopenia and/or neutropenia in participants treated with ofatumumab	Proportion of participants on ofatumumab who experienced ⩾1 infection within 1 month prior and until 1 month after IgM < LLN was 31.1% (52/167; 2 serious) vs. 51.5% (400/777) with IgM ⩾ LLN (18 serious) Overall, 1/11 participants with concurrent IgM < LLN and lymphopenia and/or neutropenia No association was observed with decreased Ig levels and increased risk of serious/nonserious infections or infections in conjunction with lymphopenia and/or neutropenia in participants treated with ofatumumab
ASCLEPIOS I/II trials [31], multinational		At week 120, no participants reached IgG levels < 50% LLN with ofatumumab (ASCLEPIOS I and II, median[g/L]: 10.57 and 9.57, respectively) or teriflunomide (10.01 and 9.65) The proportion of participants who experienced infections after the first drop of IgG levels below LLN was numerically higher for ofatumumab (45.5%) versus teriflunomide (36.4%). All infections were Grade 1/2, except 1 event in ofatumumab group (bilateral pneumonia, Grade 3) and 2 events in teriflunomide (pneumonia influenza and osteomyelitis, Grade 3)	Proportion of participants with IgM levels < 50% LLN was 2.1% (*n* = 20/944) for ofatumumab (median [g/L]: 0.91 and 0.59) and 0.6% (*n* = 6/933) for teriflunomide (0.84 and 0.92) at week 120. Of these, 5 ofatumumab-treated participants experienced infections, mostly nonserious (Grade 1/2), except 1 recurrent urinary tract infection (Grade 3); all infections were resolved. One participant on teriflunomide who experienced nasopharyngitis had not recovered at the time of last follow-up
ASCLEPIOS I/II trials [33], multinational		NR	During the RMS phase 3 clinical studies, decrease in mean value of immunoglobulin M (IgM) (30.9% decrease after 48 weeks and 38.8% decrease after 96 weeks) was observed and no association with risk of infections, including serious infections, was shown
ASCLEPIOS I/II trials. [42], multinational			A decrease in the mean level of IgM was observed in ofatumumab-treated participants but was not associated with an increased risk of infections
OBOE [18], Multinational	NR	NR	NR
VELOCE [37], US and Canada	NR	NR	NR
OPERA I/II: OPERA I/II [35, 36], multinational	Data were pooled for RMS and PPMS: Five serious infections occurred during a drop in IgA levels < LLN (5 AEs/215.8 PY; 2.3/100 PY) vs. IgA levels ⩾ LLN (200 AEs/9038.7 PY; 2.21/100 PY) Rates of serious infections per 100 PY during IgA < LLN = 166 episodes (127 participants), 256 PY, serious infections = 7 Rates of serious infections per 100 PY during IgA ≥ LLN = 2,131 episodes (1,965 participants) 9,726 PY, serious infections = 215	Data were pooled for RMS and PPMS: Overall, 14 serious infections occurred during a drop in IgG levels < LLN (14 AEs per 215.5 PY, equating to a rate of 6.50 AEs/100 PY), compared with IgG levels ⩾ LLN, (191 AEs/9,049.1 PY; 2.11/100 PY) Rates of serious infections per 100 PY during IgG < LLN = 288 episodes (152 participants), 255 PY, serious infections = 14. Rates of serious infections per 100 PY during IgG ≥ LLN = 2,269 episodes (1,940 participants) 9,737 PY, serious infections = 208	Data were pooled for RMS and PPMS: A total of 64 serious infections occurred during a drop in IgM < LLN (64 AEs/1,749.1 PY; 3.66/100 PY) vs. IgM levels ⩾ LLN (141 AEs/7,515.6 PY; 1.88/100 PY) Rates of serious infections per 100 PY during IgM < LLN = 929 episodes (729 participants), 2,003 PY, serious infections = 71 Rates of serious infections per 100 PY during IgM ≥ LLN = 2,368 episodes (1,383 participants) 7,989 PY, SI = 151
OPERA I/II [37], multinational	NR	Serious infections rates per 100 participant-years (95% CI) were: Q1, 1.63 (0.95–2.61); Q2, 1.55 (0.90–2.48); Q3, 1.51 (0.86–2.45); Q4, 1.11 (0.57–1.94) by baseline IgG quartiles	NR
OPERA I/II [40], multinational	Data were pooled for RMS and PPMS: For 61 participants with IgA < LLN (170.1 PY), there were 125 infections (73.48 per 100 PY, 95% CI = 61.16–87.55), and 8 serious infections (4.7 per 100 PY, 95% CI = 2.03–9.27)	Data were pooled for RMS and PPMS: For 121 participants with IgG < LLN (410.2 PY), there were 285 infections (69.48 per 100 PY, 95% CI = 61.65–78.04), and 14 serious infections (3.41 per 100 PY, 95% CI = 1.87–5.73)	Data were pooled for RMS and PPMS: For 426 participants with IgM < LLN (1,190.8 PY), there were 895 infections ( 75.16 per 100 PY, 95% CI = 70.31–80.25), and 36 serious infections (3.02 per 100 PY, 95% CI = 2,12–4.19)
OPERA I/II [43], multinational	NR	The pooled data of ocrelizumab clinical studies (RMS and PPMS) and their open-label extensions (up to approximately 7 years of exposure) have shown an association between decreased levels of immunoglobulin G (IgG < LLN) and increased rates of serious infections. The type, severity, latency, duration, and outcome of serious infections observed during episodes of Igs below LLN were consistent with the overall serious infections observed in participants treated with ocrelizumab	NR
OMS115102 [21], multinational	NR	NR	NR
NCT00676715 [22], multinational	NR	NR	NR
ASCLEPIOS I/II, APLIOS, APOLITOS, ALITHIOS [17], multinational	NR	No apparent association was observed between low IgG levels and risk of serious infections after 3.5 years of ofatumumab treatment; none of these participants with a serious infection suffered a recurrence. No COVID-19 infections were observed related to IgG < LLN during this period 1/30 (vs. 55/1,936 > LLN) participants had serious infections occurring up to 1 month prior and 1 month after any drop in IgG levels < LLN < LLN (N = 30): Participants with ≥ 1 serious infection = 1 (3.3); IR 7.02 Herpes zoster = 0 URTI = 0 UTI = 0 Pneumonia = 1 (3.3); IR 7.02 ≥ LLN (*N* = 1,936): Participants with ≥ 1 serious infection = 55 (2.8); IR 1.34 Herpes zoster = 1 (0.1); IR 0.02 URTI = 1 (0.1); IR 0.02 UTI = 6 (0.3); IR 0.14 Pneumonia = 8 (0.4); IR 0.19	No apparent association was observed between low IgM levels and risk of serious infections after 3.5 years of ofatumumab treatment; none of these participants with a serious infection suffered a recurrence. No COVID-19 infections were observed related to IgM < LLN during this period 3/454 (vs. 44/1512 > LLN) participants had serious infections occurring up to 1 month prior and 1 month after any drop in IgM levels < LLN < LLN (*N* = 454): Participants with ≥ 1 serious infection = 3 (0.7); IR 0.80 Herpes zoster = 1 (0.2); IR 0.27 URTI = 1 (0.2); IR 0.27 UTI = 1 (0.2); IR 0.27 Pneumonia = 0 ≥ LLN (*N* = 1,512): Participants with ≥ 1 serious infection = 44 (2.9); IR 1.38 Herpes zoster = 0 URTI = 0 UTI = 3 (0.2); IR 0.09 Pneumonia = 8 (0.5); IR 0.25

*AE*, adverse event; *CI*, confidence interval; *COVID*-*19*, coronavirus disease 2019; *EMA*, European Medicines Agency; *FDA*, Food and Drug Administration; *Ig*, immunoglobulin; *IR*, incidence rate; *LLN*, lower limit of normal; *NR*, not reported; *PPMS*, primary progressive multiple sclerosis; *PY*, person-years; *RMS*, relapsing multiple sclerosis; *SI*, serious infection; *SPC*, summary of product characteristics; *URTI*, upper respiratory tract infection; *US*, United States; *UTI*, urinary tract infection

Lopez Ruiz et al. [28] reported that no correlation between infection and Ig levels was found; however, the retrospective study may have had limited sample size to detect an association with 52 participants, with 7 experiencing infections, and Ig levels were reported for only 31 participants at baseline. None of the other included RWE studies reported data on the association of Ig levels with infection.

## Discussion

This SLR aimed to identify data on Ig levels over time in people with RMS treated with ocrelizumab and ofatumumab, as well as the associations between Ig levels and infection risk, and the differences therein between those treated with ocrelizumab and ofatumumab in clinical trials and also RWE. Of the 30 publications included in the review, 24 reported on clinical trials (11 trials with results for 9 trial populations), and 6 reported on RWE studies. Ocrelizumab was the treatment of interest in 4 of the 9 trial populations and in all 5 of the RWE studies; ofatumumab was the treatment of interest in 5 of the 9 trial populations identified but none of the RWE studies. This discrepancy in the number of RWE studies included was to be expected because ocrelizumab was first approved in the US in 2017 and in Europe in 2018 and has been in widespread use since [1, 44], whereas ofatumumab was approved more recently, in 2020 and 2021, respectively, for treatment of RMS [6, 33]. A third anti-CD20 monoclonal antibody, ublituximab, was also in development for the treatment of RMS at the time of performing this SLR, but not yet approved and therefore not included in this review.

In trials and RWE studies evaluating ocrelizumab, IgG levels decreased in most studies identified. For instance, in the OPERA I and II trials, a decrease in mean IgG levels was observed in the ocrelizumab treatment arm, as well as after a switch to ocrelizumab in those who had been originally treated with IFN β [38]. Over a period of up to 7 years, serum IgG levels decreased at an average rate of 0.33 g/L per year (− 3% per year). At 312 weeks, IgG levels fell below the LLN in 7.7% of participants [38]. Mean IgG levels were generally stable in the VELOCE trial, with some fluctuation over time, and decreased slightly at 24 weeks from baseline (although it should be noted that this vaccine response study had a much shorter follow-up duration of only 24 weeks) [19]. Similar, albeit less consistent, patterns were also observed in the RWE studies: whereas most studies observed overall decreases in IgG levels over time with ocrelizumab treatment [25, 26, 28], some studies observed stable IgG levels or even slight increases in IgG levels from baseline [23, 24]. IgM levels appeared to decrease with ocrelizumab treatment. In the 1 ocrelizumab trial population for which IgA levels were reported, decreases in IgA levels were observed with ocrelizumab treatment and, interestingly, increasing IgA levels with IFN treatment [38]. In a retrospective RWE study, serum IgA levels were reportedly not affected by 52 weeks of treatment with ocrelizumab; however, no specific IgA values were provided.

Among ofatumumab trials, a transient reduction in IgG levels from baseline was observed in the ASCLEPIOS I and II trials until week 48, which stabilized by the end of follow-up [41]. When ASCLEPIOS I and II were further pooled with APOLITOS, APLIOS, and ALITHIOS, IgG levels also remained stable with up to 3.5 years of ofatumumab treatment [17]. These observations suggest that, in general, IgG levels do not diminish overall during ongoing treatment with ofatumumab in RMS, whereas IgG levels may be more likely to reduce over time with other B cell–depleting therapies, such as with ocrelizumab treatment. Among ofatumumab trials, a pattern of decreasing IgM levels with ofatumumab treatment was also generally observed, although most participants’ levels remained above LLN. In a dose-ranging study of intravenous ofatumumab, IgA remained stable [21].

Very few studies in people with RMS have reported an association between Ig levels with infection risk, in particular with respect to serious infections (which may have a more substantial impact on patients and on healthcare utilization). In the OPERA I/II trials, a numerical trend of lower rates of serious infections among higher quartiles of baseline IgG level was observed. Furthermore, when OPERA I/II data were pooled with those from the ORATORIO trial in PPMS, an association was observed between decreased IgG, IgM, and IgA levels and an increased risk of serious infection in ocrelizumab-treated participants, which was strongest for IgG and less pronounced for IgM or IgA [36]. It is to be noted that these data include participants who received any dose of ocrelizumab during the controlled treatment and associated open-label extension periods of the phase 3 OPERA and ORATORIO studies. Furthermore, these results must be interpreted cautiously given that they were observed in a combined population of people with RMS and people with PPMS, who in general tend to be older than those with RMS. The effect of age as a potential confounder affecting the relationship between Ig levels and infections may not be adequately accounted for. In the ASCLEPIOS I/II trials, no clear association was observed with decreased IgM or IgG levels and increased risk of serious/nonserious infections in participants treated with ofatumumab [6, 29]. Similarly, pooled analyses from the ASCLEPIOS I/II, APLIOS, APOLITOS, and ALITHIOS trials concluded that there was no apparent association between low IgG or IgM levels with risk of serious infection after 3.5 years of treatment with ofatumumab [17]; 4-year data from ALITHIOS presented after this SLR was conducted have shown consistent results [13]. No further information on the association of Ig levels with infection was reported across the remaining trials. Among real-world studies, Lopez Ruiz et al. [28] reported that no correlation between infection and Ig levels was found with ocrelizumab treatment. However, this retrospective study was small, potentially underpowered, very few people actually experienced infections, and Ig levels were only reported for a subset of the study cohort. Aside from this RWE study, none of the included RWE studies reported data on the association of Ig levels with infection.

Taken together, the results of this SLR suggest that ofatumumab therapy for people with RMS may have a more favorable effect than ocrelizumab therapy on IgG levels over time as observed in clinical trials, although it is important to note that no head-to-head trials have been conducted for these therapies. IgM decreases were seen with both ofatumumab and ocrelizumab therapy. There are a number of potential mechanisms for differences in IgG effects with ofatumumab and ocrelizumab. First, ofatumumab and other anti-CD20 monoclonal antibodies show differences in B cell depletion due to variations in epitope binding, avidity, and off rate [8, 45]. Ofatumumab, for example, binds to a unique CD20 epitope, attaching closer to the cell membrane than other monoclonal antibodies, potentially accounting for greater complement-dependent cytotoxicity and B cell lysis [8]. In addition, anti-CD20 monoclonal antibodies may have different biologic effects based on mechanism of action and route of administration (e.g., with ofatumumab administered at a lower dose and more frequently than ocrelizumab, and via subcutaneous injection), in turn leading to different subsets of B cells depleted and variations in time to B cell repletion [8].

It is important to consider these results within the context of the evidence base. The total duration of treatment was shorter for ofatumumab trials compared with ocrelizumab trials. Because IgG values decline very slowly over time, it is possible that an effect of ofatumumab on IgG has not yet been detected in trials. In addition, ofatumumab trials included a mandated treatment interruption for notably low IgG levels (> 20% below LLN) and notably low IgM levels (> 10% below LLN) [9], whereas in the ocrelizumab trials, treatment interruptions or discontinuations occurred based on low IgG levels but not low IgM levels. In the ofatumumab trials, the proportions of patients with treatment interruptions and discontinuations, respectively, were 0.1% and 0.2% for IgG levels and 9.1% and 3.3% for IgM levels [17]. Although in theory this might obscure potential IgG hypogammaglobinemia in the phase 3 ofatumumab core and extension trials, it is important to note that, for the majority of ofatumumab-treated patients, treatment interruptions were brief (< 2 months), potentially suggesting that there is a plausible biologic difference in effect of ofatumumab and ocrelizumab on IgG levels. Furthermore, results from 2 ocrelizumab trial populations, NCT00676715 [22] and OPERA I/II [38], respectively indicated a decrease in mean IgG level for ocrelizumab-treated patients at week 120 and increasing proportions of ocrelizumab-treated patients with IgG < LLN over time, up to week 168—the time points most comparable to those reported in the ofatumumab trials.

Importantly, the results of the RWE studies must be interpreted with caution; these studies are limited by smaller sample sizes, cohort differences, and heterogeneous testing methods, potentially contributing to the inconsistency in IgG observed across these studies as compared with clinical trial data. Furthermore, no RWE studies were identified for ofatumumab, and the evidence may evolve as such studies are conducted. In addition, lack of reporting of IgA levels across studies highlights a clear gap in knowledge that should be addressed in larger, future longitudinal studies. Few studies specified the types of infections they monitored, which may result in between-study variation due to differences in the incidence of certain types of infection across regions, pathogenicity of different infectious agents, and heterogenous, individual immune responses. These studies also did not report data on the evolution of infections or the stages of the primary and secondary immune responses. For instance, IgM provides an initial short-term response to a new infection before the onset of an IgG response, and IgA is more relevant in mucosal areas [46–50].

Limited evaluations of potential associations between Ig levels with infections and with serious infections, particularly among real-world studies, additionally highlights a further gap in the understanding and clinical relevance of Ig changes in the setting of B cell–depleting therapies among people with RMS. Data on the associations between Ig levels and infection for the OPERA I/II trials evaluating ocrelizumab were pooled with data from the ORATORIO trial, which included people with PPMS, and therefore are not comparable with results from trials conducted with RMS-only populations. Treatments with regulatory approval for RMS in the US and Europe at the time of this review were our focus; studies related to treatments that are used off label in clinical practice (e.g., rituximab) or that are not yet approved (e.g., ublituximab) were not evaluated. Recent RWE has shown that treatment with rituximab is associated with increased risk of hospitalization for COVID-19 relative to other DMTs [7]. There is a need for additional RWE on the relationships between Ig levels and rates and severity of infection for those with RMS and the impact of different treatments.

Some limitations of this review must be considered in the interpretation of our study findings. Because this study is an SLR, the methodology does not support cross-study comparisons; indirect comparison of aggregate data was not feasible owing to heterogeneity in the studies and a lack of common comparators, and therefore, we present only a narrative summary of the study results. While robust SLR methods were used in this review, and multiple databases and gray literature sources were searched, double screening was not used. Quality assessments were not conducted in this review and so no conclusions can be drawn on the quality of the studies reporting data. Some of the included publications were conference abstracts/posters and, therefore, reported limited detail. Additionally, some characteristics of the real-world studies, including small sample sizes, short durations of follow-up, and inconsistent definitions of infections, limit comparisons between these studies and the conclusions that can be drawn from them.

## Conclusions

Results of this SLR suggest that in people with RMS, ofatumumab treatment might have a more favorable impact on IgG levels over time than ocrelizumab therapy, potentially as a result of these treatments’ respective mechanism of action and route of administration. There may therefore be an infection benefit risk associated with ofatumumab over ocrelizumab that warrants further study. IgM levels generally decrease with both ocrelizumab and ofatumumab treatment. Evidence from clinical trials indicates that Ig levels, and particularly IgG levels, may affect risk of infection in PwMS treated with ocrelizumab, ofatumumab, and other disease-modifying drugs. Additional long-term data are needed to further explore these findings.

## Supplementary Information

Below is the link to the electronic supplementary material.

Supplementary file1 (DOCX 110 KB)

Supplementary file2 (DOCX 158 KB)
