# Supplementary material for: Systematic literature review of immunoglobulin trends for anti-CD20 monoclonal antibodies in multiple sclerosis
Source: Neurol Sci. 2023 Jan 17;44(5):1515–32. doi: 10.1007/s10072-022-06582-y (PMC9843103; doi:10.1007/s10072-022-06582-y)
Supplement: Supplementary file 1 — Supplementary file1 (DOCX 110 KB) [file 10072_2022_6582_MOESM1_ESM.docx]

Table S1. Study Design of Trials Included in the Systematic Literature Review

| Trial name, primary author (year), country | Study design | Sponsor/ institute | Population | Inclusion criteria | Exclusion criteria | Treatment | Treatment schedule and dosing | Concomitant treatments |
| --- | --- | --- | --- | --- | --- | --- | --- | --- |
| ASCLEPIOS I/II trials, Hauser et al. (2020), Multinational | Phase 3 RCT, multicenter, double-blind | Novartis | Patients with RMS | Patients with RMS in the phase 3 ASCLEPIOS I/II trials, age of 18-55 years; a diagnosis of MS (according to the 2010 revised McDonald criteria) with a relapsing-remitting course or a secondary progressive course with disease activity (according to the criteria of Lublin et al.); an EDSS score of 0-5.5; at least 1 relapse in the year before screening, at least 2 relapses in the 2 years before screening, or at least 1 lesion detected with the use of gadolinium enhancement on MRI in the year before randomization; a neurologically stable condition for at least 1 month before randomization. | PPMS or SPMS without disease activity; meeting the criteria for neuromyelitis optica; disease duration of more than 10 years with an EDSS score of 2.0 or less; pregnant or lactating; woman of childbearing potential unless using highly effective methods of contraception during the trial and for 12 months postdosing; sexually active male unless using condoms during intercourse while receiving trial drug; active, chronic immune system disease other than MS or having an immunodeficiency syndrome; neurological findings consistent with PML or confirmed PML; active systemic infections or acquired immunodeficiency syndrome or testing positive for HIV antibody at screening; at risk of developing or having reactivation of hepatitis, syphilis or tuberculosis; received any live or live attenuated vaccines during the 2 months before randomization; been treated with medications as specified or within timeframes; any other disease or condition that could interfere with participation in the trial. | Ofatumumab vs. Teriflunomide | Ofatumumab 20 mg (initial doses: Days 1, 7, and 14; subsequent doses: every 4 weeks from week 4 onwards). Oral teriflunomide 14 mg once-daily for up to 30 months (m, mean follow-up duration: 18 months). | NR |
| OBOE, Weber et al. (2020), Multinational  [abstract and presentation] | Phase 4 open-label, multicenter RCT | Genentech, Inc. | 79 of 100 total patients with RMS with available CSF samples. | General inclusion criteria:  For women of childbearing potential: agreement to remain abstinent or use contraceptive methods.  Specific to RMS participants: Diagnosis of RMS in accordance with the 2010 revised McDonald criteria; EDSS score of 0-5.5 points at screening; disease duration from the onset of MS symptoms < 15 years in participants with an EDSS score > 5.0 at screening; treatment-naive or receiving treatment with DMTs; at least 1 clinically documented relapse in the past year and/or at least 1 T1-weighted Gd-enhancing lesion in the past year and/or at least 1 new T2 lesion in the past year at the time of enrollment.  Specific to RMS cohort arm 4:  Must meet inclusion criteria for the RMS cohort; willing to remain on the same dose and regimen of current standard of care, or no treatment if treatment naive, for 12 weeks after study.  Specific to PPMS Participants:  PPMS in accordance with the 2010 revised McDonald criteria; EDSS score of 3.0-6.5, at screening; disease duration from the onset of MS symptoms < 10 years in participants with an EDSS at screening ≤ 5.0; history of either elevated IgG Index or 1 or more IgG oligoclonal bands detected by isoelectric focusing. | SPMS without relapses for at least 1 year; history or known presence of recurrent or chronic infection; history of recurrent aspiration pneumonia requiring antibiotic therapy; history of cancer; history of or currently active primary or secondary immunodeficiency; history of coagulation disorders; history of severe allergic or anaphylactic reactions to humanized or murine monoclonal antibodies; history of alcohol or other drug abuse within 24 weeks prior to enrollment; known presence or history of other neurologic disorders; significant, uncontrolled disease; congestive heart failure; known active bacterial, viral, fungal, mycobacterial infection, or any major episode of infection requiring hospitalization or treatment with IV antibiotics; any concomitant disease that may require chronic treatment with systemic corticosteroids or immunosuppressants during the course of the study; contraindications or intolerance to oral or IV corticosteroids, including IV methylprednisolone, according to the country label; contraindication for LP; previous treatment with B-cell–targeted therapies; previous treatment with natalizumab, alemtuzumab, anti-CD4 agents, cladribine, teriflunomide, cyclophosphamide, mitoxantrone, azathioprine, mycophenolate mofetil, cyclosporine, methotrexate, total body irradiation, or bone marrow transplantation; treatment with fingolimod, dimethyl fumarate, or similar treatment within 6 months prior to enrollment; receipt of a live vaccine within 6 weeks prior to enrollment; systemic corticosteroid therapy within 4 weeks prior to baseline; previous or concurrent treatment with any investigational agent or treatment with any experimental procedure for multiple sclerosis; certain laboratory abnormalities or findings at screening; inability to complete an MRI; lack of peripheral venous access; pregnant or lactating, or intending to become pregnant during the study.  Specific to RMS participants:  Diagnosis of PPMS or SPMS without relapses. | Ocrelizumab + lumbar puncture | For patients with RMS: Ocrelizumab as two 300-mg IV infusion on Days 1 and 15 then as single infusion of 600 mg on Weeks 24 and 48. Participants will receive an LP before the start of dosing (Week 1, treatment baseline) with ocrelizumab and a second LP at Week 12. Participants will be asked to have an additional optional LP at Week 12, 24, or 52. Participants that complete the study and continue to receive ocrelizumab will receive single infusions every 24 weeks starting from Week 72. | Participants will receive 100 mg of IV methylprednisolone (or an equivalent) prior to ocrelizumab infusion. Participants will receive an antihistamine, such as diphenhydramine, prior to ocrelizumab infusion. |
| VELOCE, Bar-Or et al. (2020), US & Canada | Phase 3 open-label, multicenter RCT | Hoffmann-La Roche | Adult patients with RRMS | Adult patients (18-55 years old) with RMS in accordance with the revised McDonald criteria, (baseline Expanded Disability Status Scale score 0-5.5) who received ≥ 1 TT-containing vaccination (tetanus and diphtheria or tetanus, diphtheria, and acellular pertussis) > 2 years before screening were enrolled across 19 centers in the United States and 2 centers in Canada between October 2015 and August 2016. For sexually active female participants of reproductive potential, use of reliable means of contraception. | Positive serum β-human chorionic gonadotropin tests at screening; prior immunization with TT-containing vaccine within 2 years of screening, 23-valent 23-PPV within 5 years of screening, World Health Organization– recommended 2015/2016 or 2016/2017 seasonal influenza vaccine for the Northern Hemisphere, or keyhole limpet hemocyanin; prior treatment with B-cell–targeted therapies, lymphocyte-trafficking blockers, alemtuzumab, anti-CD4, cladribine, cyclophosphamide, mitoxantrone, azathioprine, mycophenolate mofetil, cyclosporine, methotrexate, total body irradiation, or bone marrow transplantation; or levels of serum IgG ≥ 18% below the LLN (< 4.6 g/L) or levels of serum IgM ≥ 8% below the LLN (< 0.37 g/L). CT.gov: Contraindications for or intolerance to oral or IV corticosteroids, including IV methylprednisolone, according to the country label; known presence of other neurologic disorders; treatment with any investigational agent within 24 weeks of screening or 5 half-lives of the investigational drug, whichever is longer, or treatment with any experimental procedure for MS. | Ocrelizumab vs. control | Eligible patients were randomized (2:1) either to OCR (administered as two 300-mg IV infusions separated by 14 days) or to a control group in which patients either continued their current IFN-beta therapy (allowed owing to the reported absence of effects on vaccine responses 6-9) or received no disease-modifying treatment. Patients randomized to OCR were further divided into 2 groups: OCR1 (patients received additional 13-PCV booster for 23-PPV) and OCR2 (patients received seasonal influenza vaccine containing 3 or 4 of the 5 strains studied. | All received tetanus toxoid-containing vaccine, Pneumovax (23-valent pneumococcal polysaccharide vaccine), and keyhole limpet hemocyanin. The OCR1 group received Prevnar (13-valent conjugate pneumococcal vaccine) 4 weeks after 23-PPV; the OCR2 and control groups received influenza vaccine. Vaccinations started 12 weeks after OCR initiation (OCR group) or on Day 1 (control group). |
| OPERA I/II, Hauser et al. (2017), Multinational | Phase 3 multicenter, double-blind RCT | Hoffmann-La Roche | RMS | 18-55 years; a diagnosis of MS (according to the 2010 revised McDonald criteria); an EDSS score of 0-5.5 at screening; at least 2 documented clinical relapses within the previous 2 years or 1 clinical relapse within the year before screening; MRI of the brain showing abnormalities consistent with MS; and no neurologic worsening for at least 30 days before both screening and baseline (Day 1 trial visit). Patients who meet the following entry criteria may participate in the OLE phase:  Complete the 96-week, double-blind, double-dummy treatment period, and who in the opinion of the Investigator may benefit from treatment with ocrelizumab; are able and willing to provide written informed consent for the OLE phase and to comply with the study protocol; are willing to continue to use at least 2 contraceptive methods; meet retreatment criteria with ocrelizumab. | Diagnosis of PPMS, previous treatment with any B-cell–targeted therapy or other immunosuppressive medication, disease duration of more than 10 years in combination with an EDSS score of 2.0 or less at screening. Contraindications for MRI; known presence of other neurological disorders which may mimic MS; pregnancy or lactation; requirement for chronic treatment with systemic corticosteroids or immunosuppressants during the study; history of or currently active primary or secondary immunodeficiency; history of severe allergic or anaphylactic reactions to humanized or murine monoclonal antibodies; active infection, or history of or known presence of recurrent or chronic infection; history of PML; contraindications to or intolerance of oral or iv corticosteroids; contraindications to Rebif or incompatibility with Rebif use. | Ocrelizumab vs. Interferon beta-1a | Patients were randomly assigned, in a 1:1 ratio, to receive ocrelizumab 600 mg by means of intravenous infusion every 24 weeks, administered as two 300-mg infusions on Days 1 and 15 for the first dose and as a single 600-mg infusion thereafter, or interferon beta-1a at a dose of 44 μg, administered subcutaneously 3 times weekly throughout the 96-week treatment period.  OLE: Patients who received OCR in the DBP continued OCR and patients from the IFN-β-1a group were switched to OCR, given every 24 weeks; in order to maintain blinding and in accordance with the start of the DBP, all patients received the first dose of OCR as 2 separate 300 mg infusions, 2 weeks apart. | All the patients received one 100-mg dose of intravenous methylprednisolone before each infusion. Prophylaxis with analgesic or antipyretic agents and an antihistamine was recommended, but the decision to use these medications was left up to the infusion center. |
| OMS115102, NCT (2008), Multinational | Phase 2 multicenter, double-blind, crossover, dose-finding RCT | GlaxoSmithKline | RRMS | 18-55 years of age with definite diagnosis of RRMS according to McDonald criteria; at least 2 confirmed relapses within the last 24 months or at least 1 confirmed relapse within the last 12 months or 1 confirmed relapse between 12 and 24 months prior to screening, and at least 1 documented T1 Gd-enhancing lesion on an MRI performed within 12 months prior to screening; patients with disability equivalent to EDSS score of 0-5.0 at screening; neurologically stable patients with no evidence of relapse for at least 30 days prior to start of screening and during the screening phase; female patients must be either postmenopausal, surgically incapable of bearing children or practicing an acceptable method of birth control; females of childbearing potential must have a negative pregnancy test at screening visit prior to entry into the treatment period. | SPMS, PPMS or PRMS, or Neuromyelitis optica; PML; MRI scan indicating any other clinically significant brain abnormality other than MS; patients unable to undergo MRI scans or who lack adequate peripheral venous access; patients who have had the following treatments:  lymphocyte-depleting therapies, anti-CD4, cladribine, total body irradiation, bone marrow transplantation, mitoxantrone or cyclophosphamide, anti-CD20 treatments or any monoclonal antibodies; immunoglobulin, azathioprine, cyclosporine, tacrolimus or other immunosuppressive agents, immunomodulatory agents or plasma exchange within 6 months prior to randomization in the trial apart from Glatiramer Acetate and IFN-b; glucocorticoids or ACTH within 1 month prior to the screening in the trial; a live vaccine within 1 month prior to screening; plasmapheresis for treatment of relapses within 2 months prior to randomization; therapy with statins or HRT within 1 month or less prior to screening; past or current history of medically significant adverse effects from Cetirizine, Prednisolone, Paracetamol/acetaminophen; plasma proteins or a known hypersensitivity to components of the investigational product; past or current malignancy; cancer diagnoses with a complete response of a duration of > 5 years; clinically significant cardiac disease; ECG showing significant abnormality; significant concurrent, uncontrolled medical condition; history of severe, clinically significant CNS trauma or a history or presence of myelopathy due to spinal cord compression by disk or vertebral disease; chronic or ongoing active infectious disease requiring systemic treatment; female patients who are pregnant or nursing; use of an investigational drug or other experimental therapy for a condition other than MS within 4 weeks prior to screening; serum vitamin B12 below LLN; PCR screening for JC virus; serologic evidence of hepatitis B; positive serology for HIV | Ofatumumab vs. placebo | Experimental: Cohort 1.1: 100 mg ofatumumab then placebo. Experimental: Cohort 1.2: placebo then 100 mg ofatumumab. Experimental: Cohort 2.1: 300 mg ofatumumab then placebo. Experimental: Cohort 2.2: placebo then 300 mg ofatumumab. Experimental: Cohort 3.1: 700 mg ofatumumab then placebo. Experimental: Cohort 3.2: placebo then 700 mg ofatumumab | NR |
| NCT00676715, Baker et al. (2020), Multinational | Phase 2 multicenter, parallel-group, double-blind RCT | Genentech, Inc. | RRMS | The requirement to have had 2 documented relapses or ≥ 1 relapse and ≥ 6 T2 lesions within the year prior to screening and to have an EDSS of 1-6; ability to provide written informed consent and to be compliant with the schedule of protocol assessment; RRMS); ages 18-55 years inclusive; for sexually active female and male participants of reproductive potential, use of reliable means of contraception. | SPMS or PPMS; Incompatibility with MRI; Contraindications to or intolerance of oral or IV corticosteroids; Known presence of other neurologic disorders; Pregnancy or lactation; lack of peripheral venous access; history of severe allergic or anaphylactic reactions to humanized or murine monoclonal antibodies; significant, uncontrolled disease; known active bacterial, viral, fungal, mycobacterial infection or other infection or any major episode of infection requiring hospitalization or treatment with IV antibiotics within 4 weeks prior to screening or oral antibiotics within 2 weeks prior to screening; history or known presence of recurrent or chronic infection; history of cancer; history of alcohol or drug abuse within 24 weeks prior to randomization; history of or currently active primary or secondary immunodeficiency; history of coagulation disorders; treatment with any investigational agent within 4 weeks of screening; a live vaccine within 6 weeks prior to randomization; incompatibility with Avonex use; previous treatment with rituximab; previous treatment with lymphocyte-depleting therapies except mitoxantrone; treatment with lymphocyte-trafficking blockers within 24 weeks prior to randomization; treatment with beta interferons, glatiramer acetate, IV immunoglobulin, plasmapheresis, or immunosuppressive therapies within 12 weeks prior to randomization; systemic corticosteroid therapy within 4 weeks prior to randomization. | Ocrelizumab vs. placebo vs. Avonex | People with MS were randomized to either: (a) 2 placebo intravenous infusions at 15-day intervals (b) 2 infusions of 300 mg ocrelizumab at 15-day intervals (600 mg dose), with infusion reaction prophylaxis, and (c) open-label 30 μg IFNβ administered twice a week. An additional group of people received 2 doses of 1,000 mg ocrelizumab. After 24 weeks, all groups (a-c) received ocrelizumab, which was administered at Weeks 24, 48, and 72. People treated with placebo and IFNβ initiated their ocrelizumab treatment with 2 doses of 300 mg ocrelizumab; all subsequent doses were single infusions of 600 mg at 6-month intervals. | NR |
| ASCLEPIOS I/II, APLIOS, APOLITOS, ALITHIOS, Wiendl et al. (2021), multinational | ASCLEPIOS I/II (phase 3 RCT), APOLITOS (phase 2 RCT), APLIOS (phase 2 RCT), (Long term) ALITHIOS (phase 3 open-label, single-arm, multicenter extension study) | Novartis Pharma­ceuticals | RMS | ASCLEPIOS I/II: 18-55 years of age, diagnosis of RRMS SPMS, at least 1 relapse during the previous 1 year or 2 relapses during the previous 2 years or a positive gadolinium-enhancing MRI scan in previous year, EDSS score of 0-5.5. APOLITOS: RMS, EDSS score of 0-5.5, and at least 1 appearance of a new neurological abnormality or worsening of pre-existing neurological abnormality during the previous 2 years prior to screening AND an MRI activity (Gd-enhanced T1 lesions or new or enlarging T2 lesions) in brain during the previous 1 year prior to randomization. APLIOS: Diagnosis of MS, RMS, RRMS, or SPMS course, EDSS score of 0-5.5, documentation of at least: 1 relapse during the previous year OR 2 relapses during the previous 2 years prior to screening OR a positive Gd-enhancing MRI scan during the year prior to randomization, neurologically stable within 1 month prior to randomization. ALITHIOS: Must have completed a selected Novartis MS study which dosed ofatumumab 20 mg SC every 4 weeks, written informed consent. | ASCLEPIOS I/II: PPMS, disease duration > 10 years in patients with an EDSS score of 2 or less, patients with an active chronic disease of the immune system other than MS, patients at risk of developing or having reactivation of hepatitis, patients with active systemic infections or with neurological findings consistent with PML. APOLITOS: PPMS or SPMS without disease activity, active chronic disease of the immune system other than MS, at risk of developing or having reactivation of hepatitis, active systemic infections or with neurological findings consistent with PML. APLIOS: Patients with primary progressive MS or SPMS without disease activity, disease duration of more than 10 years in patients with EDSS score of 2 or less, patients with an active chronic disease of the immune system other than MS, patients with active systemic bacterial, viral or fungal infections, or known to have AIDS or to test positive for HIV antibody at screening, patients with neurological findings consistent with PML, or confirmed PML. ALITHIOS: Emergence of any clinically significant condition/disease during the previous ofatumumab study in which study participation might result in safety risk for the subject, subjects with active systemic bacterial, viral or fungal infections, or chronic infection (e.g., AIDS), subjects taking medications prohibited by the protocol, pregnant or nursing (lactating) women. Other protocol-defined inclusion/exclusion criteria may apply. | ASCLEPIOS I/II: Ofatumumab vs. Teriflunomide, APOLITOS: ofatumumab 20 mg vs. placebo, APLIOS: ofatumumab with Pre-filled Syringe (PFS) vs. ofatumumab with Auto-Injector (AI), ALITHIOS: ofatumumab | Ofatumumab 20 mg | NR |

ACTH = adrenocorticotropic hormone; CNS = central nervous system; CSF = cerebrospinal fluid; ECG = electrocardiogram; EDSS = Expanded Disability Status Scale; HIV = human immunodeficiency viruses; HRT = hormone replacement therapy; IFN = interferon; Ig = immunoglobulin; IV = intravenous therapy; JC virus = John Cunningham virus; LLN = lower limits of normal; LP = lumbar puncture; MRI = magnetic resonance imaging; MS = multiple sclerosis; NR = not reported; OCR = ocrelizumab; OLE = open-label extension; PCR = polymerase chain reaction; PCV = pneumococcal vaccine; PML = progressive multifocal leukoencephalopathy; PPMS = primary progressive multiple sclerosis; PPV = pneumococcal polysaccharide vaccine; PRMS = progressive relapsing multiple sclerosis; RMS = relapsing multiple sclerosis; RRMS = relapsing-remitting multiple sclerosis; RCT = randomized control trial; SLR = systematic literature review; SPMS = secondary progressive multiple sclerosis; TT = tetanus toxoid; US = United States

Table S2. Baseline Characteristics of Trials Included in the Systematic Literature Review

| Trial name, author (year), country | Treatment (n) | Type of MS, n/N (%) | Age, mean (SD) | Female, n (%) | BMI, mean (SD) | Time since MS diagnosis, mean (SD) | Prior treatments, n/N (%) | Comorbidities, n/N (%) | Disability level, mean (SD) |
| --- | --- | --- | --- | --- | --- | --- | --- | --- | --- |
| ASCLEPIOS I^a^, Hauser et al. (2020), multinational | Ofatumumab (n = 465) | RMS = 438 (94.2), SPMS = 27 (5.8) | 38.9 (8.8) | 318 (68.4) | 26.21 (6.413) | 5.77 (6.05), median = 3.94 | Any interferon beta = 189 (40.6) Glatiramer acetate = 124 (26.7) Dimethyl fumarate = 36 (7.7) Teriflunomide = 8 (1.7) Daclizumab = 5 (1.1) Fingolimod = 10 (2.2) Natalizumab = 31 (6.7) Any B-cell therapy = 2 (0.4) Laquinimod = 5 (1.1)  Other DMT = 52 (11.2) No previous DMT = 191 (41.1%) | NR | EDSS score = 2.97 (1.36), median = 3 |
|  | Teriflunomide (n = 462) | RMS = 434 (93.9), SPMS = 28 (6.1) | 37.8 (9.0) | 317 (68.6) | 26.18 (6.110) | 5.64 (6.20), median = 3.49 | Any interferon beta = 193 (41.8) Glatiramer acetate = 106 (22.9) Dimethyl fumarate = 37 (8.0) Teriflunomide = 6 (1.3) Daclizumab = 12 (2.6) Fingolimod = 15 (3.2) Natalizumab = 36 (7.8) Any B-cell therapy = 3 (0.6) Laquinimod = 4 (0.9) Other DMT = 65 (14.1) No previous DMT = 182 (39.4%) |  | EDSS score = 2.94 (1.36), median = 3 |
| ASCLEPIOS II^a^, Hauser et al. (2020), multinational | Ofatumumab (n = 481) | RMS = 452 (94.0), SPMS = 29 (6.0) | 38.0 (9.3) | 319 (66.3) | 25.52 (6.009), median = 24.15 | 5.59 (6.38), median = 3.15 | Any interferon beta = 197 (41.0) Glatiramer acetate = 118 (24.5) Dimethyl fumarate = 36 (7.5) Teriflunomide = 13 (2.7) Daclizumab = 8 (1.7) Fingolimod = 13 (2.7) Natalizumab = 26 (5.4) Any B-cell therapy = 0, Laquinimod = 2 (0.4) Other DMT = 68 (14.1) No previous DMT = 195 (40.5) | NR | EDSS score = 2.90 (1.34), median = 3 |
|  | Teriflunomide (n = 474) | RMS = 450 (94.9), SPMS = 24 (5.1) | 38.2 (9.5) | 319 (67.3) | 25.69 (5.931), median = 24.53 | 5.48 (6.00), median = 3.1 | Any interferon beta = 193 (40.7) Glatiramer acetate = 149 (31.4) Dimethyl fumarate = 44 (9.3) Teriflunomide = 9 (1.9) Daclizumab = 7 (1.5) Fingolimod = 10 (2.1) Natalizumab = 20 (4.2) Any B-cell therapy = 0 Laquinimod = 7 (1.5) Other DMT = 81 (17.1) No previous disease-modifying therapy = 181 (38.2) |  | EDSS score = 2.86 (1.37), median = 2.5 |
| OBOE, Weber et al. (2020), multinational | Ocrelizumab 600 mg | RMS | NR | NR | NR | NR | NR | NR | NR |
| VELOCE, Bar-Or et al. (2020), US and Canada | Ocrelizumab 600 mg (n = 68) | RRMS | 39.7 (8.9) | 45 (66.2) | 28.9 (6.7) | 6.6 (6.6) | No prior DMT = 54% | NR | EDSS score = 2.7 (1.3) |
|  | Control (n = 34) |  | 41.4 (7.9) | 27 (79.4) | 26.6 (5.7) | 7.1 (5.2) | No prior DMT = 68% |  | EDSS score = 2.3 (1.4) |
|  | Ocrelizumab 600 mg (group A) (n = 33) |  | 40.1 (8.0) | 21 (63.6%) | NR | NR | NR |  | NR |
|  | Ocrelizumab 600 mg (group B) (n = 35) |  | 39.3 (9.7) | 24 (68.6%) | NR | NR | NR |  | NR |
| OPERA I/II double-blind period, Hauser et al. (2020), multinational | IFN β-1a for 96 weeks, switched to ocrelizumab 600 mg (n = 829) | RMS | 37.2 (9.2) | 552 (66.6) | NR | NR | NR | NR | EDSS score = 2.8 (1.3) |
|  | Ocrelizumab 600 mg (n = 827) |  | 37.1 (9.2) | 541 (65.4) |  |  |  |  | EDSS score = 2.8 (1.3) |
| OPERA I/II OLE, Hauser et al. (2020), multinational | IFN β-1a for 96 weeks, switched to ocrelizumab 600 mg (n = 623) | RMS | 39.3 (9.2) | 408 (65.5) | NR | NR | NR | NR | EDSS score = 2.7 (1.5) |
|  | Ocrelizumab 600 mg (n = 702) |  | 39.2 (9.1) | 454 (64.7) |  |  |  |  | EDSS score = 2.6 (1.3) |
| OPERA I, Hauser et al. (2017), multinational | Ocrelizumab (N = 410) | RMS | 37.1 (9.3) | 270 (65.9) | NR | 3.82 (4.80) | No prior DMT = 301/408 (73.8)  Previous DMT = 107/408 (26.2)  Interferon = 81/408 (19.9)  Glatiramer acetate = 38/408 (9.3)  Natalizumab = 0/408  Fingolimod = 1/408 (0.2)  Dimethyl fumarate = 1/408 (0.2)  Other = 2/408 (0.5) | NR | EDSS score = 2.86 (1.24) |
|  | Interferon beta-1a (N = 411) |  | 36.9 (9.3) | 272 (66.2) |  | 3.71 (4.63) | No prior DMT = 292/409 (71.4)  Previous DMT = 117/409 (28.6)  Interferon = 86/409 (21.0)  Glatiramer acetate = 37/409 (9.0)  Natalizumab = 1/409 (0.2)  Fingolimod = 0/409  Dimethyl fumarate = 0/409  Other = 0/409 |  | EDSS score = 2.75 (1.29) |
| OPERA II, Hauser et al. (2017), multinational | Ocrelizumab (N = 417) | RMS | 37.2 (9.1) | 271 (65.0) | NR | 4.15 (4.95) | No prior DMT = 304/417 (72.9)  Previous DMT = 113/417 (27.1)  Interferon = 80/417 (19.2)  Glatiramer acetate = 39/417 (9.4)  Natalizumab = 1/417 (0.2)  Fingolimod = 4/417 (1.0)  Dimethyl fumarate = 0/417  Other = 1/417 (0.2) | NR | EDSS score = 2.78 (1.30) |
|  | Interferon beta-1a (N = 418) |  | 37.4 (9.0) | 280 (67.0) |  | 4.13 (5.07) | No prior DMT = 314/417 (75.3)  Previous DMT = 103/417 (24.7)  Interferon = 75/417 (18.0)  Glatiramer acetate = 44/417 (10.6)  Natalizumab = 0/417  Fingolimod = 0/417  Dimethyl fumarate = 0/417  Other = 1/417 (0.2) |  | EDSS score = 2.84 (1.38) |
| OMS115102. Nct (2008), multinational | Ofatumumab 100 mg to Week 24 then placebo (n = 8) | RRMS | 38.0 (9.0) | 6 (75.0) | NR | NR | NR | NR | NR |
|  | Ofatumumab 300 mg to Week 24 then placebo (n = 11) |  | 36.6 (7.0) | 6 (54.5) |  |  |  |  |  |
|  | Ofatumumab 700 mg to Week 24 then placebo (n = 7) |  | 33.7 (8.4) | 4 (57.1) |  |  |  |  |  |
|  | Placebo to Week 24 then ofatumumab 100 mg (n = 4) |  | 37.0 (6.5) | 3 (75) |  |  |  |  |  |
|  | Placebo to Week 24 then ofatumumab 300 mg (n = 4) |  | 27.0 (2.2) | 3 (75) |  |  |  |  |  |
|  | Placebo to Week 24 then ofatumumab 700 mg (n = 4) |  | 44.0 (8.1) | 0 |  |  |  |  |  |
| NCT00676715, Baker et al. (2020), multinational | PBO (n = 54) | RRMS | 38.0 (8.8) | 36 (66.7) | NR | NR | NR | NR | NR |
|  | Ocrelizumab 600 mg (n = 55) |  | 35.6 (8.5) | 35 (63.6) |  |  |  |  |  |
|  | Ocrelizumab 2000 mg (n = 55) |  | 38.5 (8.7) | 38 (69.1) |  |  |  |  |  |
|  | Interferon beta-1a (Avonex) (n = 54) |  | 38.1 (9.3) | 32 (59.3) |  |  |  |  |  |
| ASCLEPIOS I/II, APLIOS, APOLITOS, ALITHIOS, Wiendl et al. (2021), multinational | NR | RMS | NR | NR | NR | NR | NR | NR | NR |

BMI = body mass index; DMT = disease-modifying therapy; EDSS = Expanded Disability Status Scale; IFN = interferon; MS = multiple sclerosis; NR = not reported; PBO = placebo; RMS = relapsing multiple sclerosis; RRMS = relapsing-remitting multiple sclerosis; SLR = systematic literature review; SPMS = secondary progressive multiple sclerosis; US = United States.

^a^ Although baseline characteristics were not available for the pooled trial, they were available for each trial separately and, as such, baseline characteristics for the pooled population could be estimated.

Table S3. Study Design of Real-world Evidence Studies Included in the Systematic Literature Review

| Trial name, author (year), country | Population | Inclusion criteria | Exclusion criteria | Treatment schedule and dosing | Concomitant treatments |
| --- | --- | --- | --- | --- | --- |
| Prezioso (2021), Italy | RRMS | Patients diagnosed with a RRMS enrolled in the study between February 2019 and May 2020 | NR | Ocrelizumab: 600 mg of ocrelizumab 2 weeks apart, followed by a single 600-mg intravenous infusion every 6 months for a total of 12 months. | NR |
| van Lierop (2021), Netherlands | RRMS and JC virus positive | Patients from the natalizumab cohort who switched to ocrelizumab because of progressive multifocal leukoencephalopathy | NR | Ocrelizumab: the first dosage of 300 mg ocrelizumab is scheduled 6 weeks after the last natalizumab dose to avoid rebound disease activity. Second 300 mg ocrelizumab dose (OCR FU1), first 600 mg dose (FU2) and second 600 mg dose (FU3). | NR |
| Edgar (2020), US  [abstract and presentation] | RRMS | Patients who initiated OCR therapy at Rocky Mountain MS Clinic by 23 July 2019, completed 2 courses of OCR therapy, and a clinic follow-up visit | NR | Ocrelizumab:  NR | NR |
| Evertsson (2020) and Evertsson (2019) [abstract and poster], US for ocrelizumab and Sweden for rituximab | RMS | The study was performed at 2 specialized MS clinics; the Karolinska University Hospital Huddinge, Sweden, and Rocky Mountain Multiple Sclerosis Clinic (RMMSC), Utah, US. The Karolinska cohort comprised all patients with RRMS SPMS initiating RTX between 2010 and 28 May 2018. The RMMSC cohort comprised all patients with RRMS or SPMS initiating OCR between 1 May 2017 and 30 November 2018. Inclusion criteria were a diagnosis of RRMS or SPMS, that treatment was initiated due to MS and that infusions had been given in intervals of 5-7 months. All patients fulfilled the revised 2017 McDonald criteria for MS, in addition the definition of SPMS relied on the 2013 Lublin criteria. | NR | Ocrelizumab or rituximab.  Ocrelizumab: two 300‑mg infusions 2 weeks apart and a single infusion of 600 mg every 5-7 months thereafter.  Rituximab: single infusion 500 or 1,000 mg of RTX, followed by a single infusion of 500 mg every 5-7 month thereafter | NR |
| Lopez Ruiz (2021), Spain [abstract and presentation] | RRMS | Patients with RRMS treated with ocrelizumab from November 2016-May 2021 at Virgen Macarena Hospital, Seville Spain. | NR | Ocrelizumab: NR | NR |

JC virus = John Cunningham Virus; MS = multiple sclerosis; NR = not reported; OCR = ocrelizumab; RMS = relapsing multiple sclerosis; RRMS = relapsing-remitting multiple sclerosis; RMMSC = Rocky Mountain Multiple Sclerosis Clinic; RTX = rituximab; SLR = systematic literature review; SPMS = secondary progressive multiple sclerosis; US = United States.

Table S4. Baseline Characteristics of Real-world Evidence Studies Included in the Systematic Literature Review

| Trial name, author (year) | Treatment (n) | Type of MS, n/N (%) | Age, mean (SD) | Female, n (%) | BMI, mean (SD) | Time since MS diagnosis, mean (SD) | Prior treatments, n/N (%) | Comorbidities, n/N (%) | Disability level, mean (SD) |
| --- | --- | --- | --- | --- | --- | --- | --- | --- | --- |
| Prezioso (2021), Italy | Ocrelizumab (n = 42) | RRMS = 42/42 (100%) | 40.34 (9.01) | 24/42 (57.1%) | NR | NR | Naive = 19  Natalizumab = 4  Others (fingolimod, dimethyl fumarate, and Aubagio) = 19 | NR | NR |
| van Lierop (2021), Netherlands | Ocrelizumab direct switch (n = 27) | RRMS | 39 (9.9) | 17/27 (63%) | NR | Median [IQR] = 13.8 [11-18] | Natalizumab = 27/27 (100%) | NR | EDSS median [IQR] = 3.0 [2.5-4.5] |
|  | Ocrelizumab indirect switch (n = 15) | RRMS | 40 (11.3) | 9/15 (56%) |  | Median [IQR] = 11.1 [9-17] | Natalizumab = 15/15 (100%)  Fingolimod = 11 Daclizumab = 3  Dimethyl fumaric acid = 1 |  | EDSS median [IQR] = 3.5 [2.5-3.5] |
| Edgar (2020), US | Ocrelizumab (n = 135) | NR | Median [IQR] = 50.5 [41-59] | 77/135 (57%) | Median [IQR] = 28.4 [24-32.8] | Median [IQR] = 13.2 [7.7-20.7] | NR | NR | NR |
|  | Ocrelizumab super response^a^ (n = 13) |  | Median [IQR] = 42 [36-56] | 8/13 (62%) | Median [IQR] = 29.7 [24.3-37.1] | Median [IQR] = 10.7 [7.2-17.7] | Tecfidera = 46.15% Tysabri = 23.08% Aubagio = 7.69%  Rituxan 23.08% |  |  |
|  | Ocrelizumab remaining population (n = 122) |  | Median [IQR] = 51 [41-59] | 69/122 (57%) | Median [IQR] = 28.4 [24-32.5] | Median [IQR] = 13.7 [7.7-20.7] | Tecfidera = 18.84% Tysabri = 44.93% Aubagio = 3.62%  Rituxan 12.32% Copaxone = 2.90% Gilenya = 0.72%  Plegridy = 1.45%  NA = 15.22% |  |  |
| Evertsson (2020) and Evertsson (2019)^b^, US | Ocrelizumab (n = 161) | RRMS = 161/161 (100%) | 49.8 (11.9) | 95/161 (59.0%) | NR | 12.5 (8.32) | Aubagio = 9/161 (5.59%) Gilenya = 1/161 (0.62%) Injectable = 14/161 (8.69%) Naive = 7/161 (4.34%) Rituxan = 23/161 (14.2%) Tecfidera = 40/161 (24.8%) Tysabri = 66/161 (40.9%) Unknown = 1/161 (0.62%) | NR | NR |
| Lopez Ruiz (2021), Spain | Ocrelizumab (n = 52) | RRMS | 39.5 (8.7) | 65.3% | NR | 11.1 (NR) | Mean (SD) previous disease-modifying therapy = 1.7 (1.1). Naive = 5/52 (9.6%) | NR | Median [IQR] = 3.5 [1.5-6.5] |
