# Supplementary material for: Systematic literature review of immunoglobulin trends for anti-CD20 monoclonal antibodies in multiple sclerosis
Source: Neurol Sci. 2023 Jan 17;44(5):1515–32. doi: 10.1007/s10072-022-06582-y (PMC9843103; doi:10.1007/s10072-022-06582-y)
Supplement: Supplementary file 2 — Supplementary file2 (DOCX 158 KB) [file 10072_2022_6582_MOESM2_ESM.docx]

## Appendix B. Detailed Outcomes Tables

Table S5. Outcomes Reported From Clinical Trials

| Trial name, author (year) | Treatment | Timepoint | Mean or median | | | Change from baseline | | | Percentage achieving a certain level | | |
| --- | --- | --- | --- | --- | --- | --- | --- | --- | --- | --- | --- |
|  |  |  | IgA | IgG | IgM | IgA | IgG | IgM | IgA | IgG | IgM |
| ASCLEPIOS I/II trials  [1-6] | Ofatumumab (ASCLEPIOS I n = 465, ASCLEPIOS II n = 481) | Overall postbaseline | NR | NR | NR | NR | NR | NR | NR | < LLN = 134/944 (14.2%). No participants reached IgG levels 50% below LLN. IgG levels 20% below the LLN were observed at least once in 1.3% of participants | < LLN = 167/944 (17.7%), < 30% LLN = 3.8%, < 50% LLN = 2.1%. IgM levels 10% below the LLN were observed at least once in 14.3% of participants |
|  | Teriflunomide (ASCLEPIOS I n = 462, ASCLEPIOS II n = 474) | Overall postbaseline | NR | NR | NR | NR | NR | NR | NR | < LLN = 214/934 (22.9%). IgG levels 20% below the LLN were observed at least once in 3.2% of participants | < LLN = 62/933 (6.6%). IgM levels 10% below the LLN were observed at least once in 4.5% of participants |
|  | Ofatumumab (ASCLEPIOS I n = 465, ASCLEPIOS II n = 481) | Week 0 | NR | Digitized data: Median (9.99) IQR (8.43-11.4) g/L. N = NR | Digitized data: Median (1.24) IQR (0.873-1.64) g/L. N = NR | NR | NR | NR | NR | NR | NR |
|  | Teriflunomide (ASCLEPIOS I n = 462, ASCLEPIOS II n = 474) | Week 0 | NR | Digitized data: Median (9.99) IQR (8.65-11.4) g/L. N = NR | Digitized data: Median (1.23) IQR (0.891-1.64) g/L. N = NR | NR | NR | NR | NR | NR | NR |
|  | Ofatumumab (ASCLEPIOS I n = 465, ASCLEPIOS II n = 481) | Week 4 | NR | Digitized data: Median (9.73) IQR (8.33-11.2) g/L. N = NR | Digitized data: Median (1.12) IQR (0.739-1.52) g/L. N = NR | NR | NR | NR | NR | NR | NR |
|  | Teriflunomide (ASCLEPIOS I n = 462, ASCLEPIOS II n = 474) | Week 4 | NR | Digitized data: Median (9.39) IQR (8.25-10.9) g/L. N = NR | Digitized data: Median (1.21) IQR (0.827-1.56) g/L. N = NR | NR | NR | NR | NR | NR | NR |
|  | Ofatumumab (ASCLEPIOS I n = 465, ASCLEPIOS II n = 481) | Week 12 | NR | Digitized data: Median (9.82) IQR (8.36-11.3) g/L. N = NR | Digitized data: Median (1.02) IQR (0.663-1.4) g/L. N = NR | NR | NR | NR | NR | NR | NR |
|  | Teriflunomide (ASCLEPIOS I n = 462, ASCLEPIOS II n = 474) | Week 12 | NR | Digitized data: Median (8.96) IQR (7.82-10.3) g/L. N = NR | Digitized data: Median (1.04) IQR (0.726-1.38) g/L. N = NR | NR | NR | NR | NR | NR | NR |
|  | Ofatumumab (ASCLEPIOS I n = 465, ASCLEPIOS II n = 481) | Week 24 | NR | Digitized data: Median (9.66) IQR (8.22-11.1) g/L. N = NR | Digitized data: Median (0.903) IQR (0.574-1.3) g/L. N = NR | NR | NR | NR | NR | NR | NR |
|  | Teriflunomide (ASCLEPIOS I n = 462, ASCLEPIOS II n = 474) | Week 24 | NR | Digitized data: Median (8.78) IQR (7.54-10.1) g/L. N = NR | Digitized data: Median (0.967) IQR (0.637-1.32) g/L. N = NR | NR | NR | NR | NR | NR | NR |
|  | Ofatumumab (ASCLEPIOS I n = 465, ASCLEPIOS II n = 481) | Week 36 | NR | Digitized data: Median (9.43) IQR (7.97-10.8) g/L. N = NR | Digitized data: Median (0.84) IQR (0.548-1.22) g/L. N = NR | NR | NR | NR | NR | NR | NR |
|  | Teriflunomide (ASCLEPIOS I n = 462, ASCLEPIOS II n = 474) | Week 36 | NR | Digitized data: Median (8.71) IQR (7.57-9.85) g/L. N = NR | Digitized data: Median (0.979) IQR (0.675-1.33) g/L. N = NR | NR | NR | NR | NR | NR | NR |
|  | Ofatumumab (ASCLEPIOS I n = 465, ASCLEPIOS II n = 481) | Week 48 | NR | Digitized data: Median (9.46) IQR (8.17-10.9) g/L. N = NR | Digitized data: Median (0.827) IQR (0.548-1.18) g/L. N = NR | NR | Mean decrease = 4.3% (−0.435 g/L) N = 824 | Mean decrease = 30.9% (−0.420 g/L), N = 824 | NR | NR | NR |
|  | Teriflunomide (ASCLEPIOS I n = 462, ASCLEPIOS II n = 474) | Week 48 | NR | Digitized data: Median (8.8) IQR (7.71-10) g/L. N = NR | Digitized data: Median (0.992) IQR (0.701-1.37) g/L. N = NR | NR | NR | NR | NR | NR | NR |
|  | Ofatumumab (ASCLEPIOS I n = 465, ASCLEPIOS II n = 481) | Week 60 | NR | Digitized data: Median (9.72) IQR (8.34-11.2) g/L. N = NR | Digitized data: Median (0.751) IQR (0.498-1.14) g/L. N = NR | NR | NR | NR | NR | NR | NR |
|  | Teriflunomide (ASCLEPIOS I n = 462, ASCLEPIOS II n = 474) | Week 60 | NR | Digitized data: Median (9.02) IQR (7.88-10.4) g/L. N = NR | Digitized data: Median (0.967) IQR (0.688-1.33) g/L. N = NR | NR | NR | NR | NR | NR | NR |
|  | Ofatumumab (ASCLEPIOS I n = 465, ASCLEPIOS II n = 481) | Week 72 | NR | Digitized data: Median (9.99) IQR (8.49-11.5) g/L. N = NR | Digitized data: Median (0.751) IQR (0.498-1.08) g/L. N = NR | NR | NR | NR | NR | NR | NR |
|  | Teriflunomide (ASCLEPIOS I n = 462, ASCLEPIOS II n = 474) | Week 72 | NR | Digitized data: Median (9.41) IQR (8.15-10.8) g/L. N = NR | Digitized data: Median (0.967) IQR (0.675-1.33) g/L. N = NR | NR | NR | NR | NR | NR | NR |
|  | Ofatumumab (ASCLEPIOS I n = 465, ASCLEPIOS II n = 481) | Week 84 | NR | Digitized data: Median (10) IQR (8.61-11.6) g/L. N = NR | Digitized data: Median (0.751) IQR (0.485-1.04) g/L. N = NR | NR | NR | NR | NR | NR | NR |
|  | Teriflunomide (ASCLEPIOS I n = 462, ASCLEPIOS II n = 474) | Week 84 | NR | Digitized data: Median (9.38) IQR (8.19-10.9) g/L. N = NR | Digitized data: Median (0.967) IQR (0.663-1.31) g/L. N = NR | NR | NR | NR | NR | NR | NR |
|  | Ofatumumab (ASCLEPIOS I n = 465, ASCLEPIOS II n = 481) | Week 96 | NR | Digitized data: Median (10.1) IQR (8.6-11.6) g/L. N = NR | Digitized data: Median (0.701) IQR (0.472-1) g/L. N = NR | NR | Mean Increase = 2.2% (+0.249 g/L), N = 304 | Mean decrease = 38.8% (−0.537 g/L), N = 304 | NR | < LLN = 9/333 (2.7%), < 20% LLN = 0 | < LLN = 37/333 (11.1%), < 10% LLN = 7.8% (26/333) |
|  | Teriflunomide (ASCLEPIOS I n = 462, ASCLEPIOS II n = 474) | Week 96 | NR | Digitized data: Median (9.84) IQR (8.54-11.1) g/L. N = NR | Digitized data: Median (0.929) IQR (0.688-1.27) g/L. N = NR | NR | NR | NR | NR | < LLN = 19/316 (6.0%), < 20% LLN = 0.3% (1/316) | < LLN = 6/316 (1.9%), < 10% LLN = 1.3% (4/316) |
|  | Ofatumumab (ASCLEPIOS I n = 465, ASCLEPIOS II n = 481) | Week 108 | NR | Digitized data: Median (10.1) IQR (8.61-11.5) g/L. N = NR | Digitized data: Median (0.726) IQR (0.460-0.992) g/L. N = NR | NR | NR | NR | NR | NR | NR |
|  | Teriflunomide (ASCLEPIOS I n = 462, ASCLEPIOS II n = 474) | Week 108 | NR | Digitized data: Median (10) IQR (8.53-11.3) g/L. N = NR | Digitized data: Median (0.916) IQR (0.713-1.33) g/L. N = NR | NR | NR | NR | NR | NR | NR |
|  | Ofatumumab (ASCLEPIOS I n = 465, ASCLEPIOS II n = 481) | Week 120 | NR | Digitized data: Median (9.74) IQR (8.61-11.7) g/L. N = NR | Digitized data: Median (0.713) IQR (0.498-1.07) g/L. N = NR | NR | NR | Decrease in IgM in 7.7% participants | NR | < 50% LLN = 0 | < 50% LLN n = 20/944 (2.1%) |
|  | Teriflunomide (ASCLEPIOS I n = 462, ASCLEPIOS II n = 474) | Week 120 | NR | Digitized data: Median (9.62) IQR (8.23-10.8) g/L. N = NR | Digitized data: Median (0.865) IQR (0.624-1.16) g/L. N = NR | NR | NR | Decrease in IgM in 3.1% participants | NR | < 50% LLN = 0 | < 50% LLN n = 6/933 (0.6%) |
| ASCLEPIOS I [2] | Ofatumumab (n = NR) | baseline | NR | Mean (10.277)^a^ g/L. N = 465 | Mean (1.348)^a^ g/L. N = 465 | NR | NR | NR | NR | NR | NR |
|  | Teriflunomide (n = NR) | baseline | NR | Mean (10.332)^a^ g/L. N = 461 | Mean (1.367)^a^ g/L. N = 461 | NR | NR | NR | NR | NR | NR |
|  | Ofatumumab (n = NR) | Week 4 | NR | Mean^a^ g/L. N = 463 | Mean^a^ g/L. N = 463 | NR | NR | NR | NR | NR | NR |
|  | Teriflunomide (n = NR) | Week 4 | NR | Mean^a^ g/L. N = 457 | Mean^a^ g/L. N = 456 | NR | NR | NR | NR | NR | NR |
|  | Ofatumumab (n = NR) | Week 12 | NR | Mean^a^ g/L. N = 458 | Mean^a^ g/L. N = 458 | NR | NR | NR | NR | NR | NR |
|  | Teriflunomide (n = NR) | Week 12 | NR | Mean^a^ g/L. N = 454 | Mean^a^ g/L. N = 453 | NR | NR | NR | NR | NR | NR |
|  | Ofatumumab (n = NR) | Week 24 | NR | Mean^a^ g/L. N = 445 | Mean^a^ g/L. N = 445 | NR | NR | NR | NR | NR | NR |
|  | Teriflunomide (n = NR) | Week 24 | NR | Mean^a^ g/L. N = 436 | Mean^a^ g/L. N = 436 | NR | NR | NR | NR | NR | NR |
|  | Ofatumumab (n = NR) | Week 36 | NR | Mean^a^ g/L. N = 432 | Mean^a^ g/L. N = 432 | NR | NR | NR | NR | NR | NR |
|  | Teriflunomide (n = NR) | Week 36 | NR | Mean^a^ g/L. N = 429 | Mean^a^ g/L. N = 429 | NR | NR | NR | NR | NR | NR |
|  | Ofatumumab (n = NR) | Week 48 | NR | Mean (9.892)^a^ g/L. N = 421 | Mean (0.934)^a^ g/L. N = 421 | NR | NR | NR | NR | NR | NR |
|  | Teriflunomide (n = NR) | Week 48 | NR | Mean (9.179)^a^ g/L. N = 415 | Mean (1.118)^a^ g/L. N = 415 | NR | NR | NR | NR | NR | NR |
|  | Ofatumumab (n = NR) | Week 60 | NR | Mean^a^ NR g/L. N = 410 | Mean^a^ NR g/L. N = 410 | NR | NR | NR | NR | NR | NR |
|  | Teriflunomide (n = NR) | Week 60 | NR | Mean^a^ g/L. N = 400 | Mean^a^ g/L. N = 400 | NR | NR | NR | NR | NR | NR |
|  | Ofatumumab (n = NR) | Week 72 | NR | Mean^a^ g/L. N = 407 | Mean^a^ g/L. N = 407 | NR | NR | NR | NR | NR | NR |
|  | Teriflunomide (n = NR) | Week 72 | NR | Mean^a^ g/L. N = 388 | Mean^a^ g/L. N = 388 | NR | NR | NR | NR | NR | NR |
|  | Ofatumumab (n = NR) | Week 84 | NR | Mean^a^ g/L. N = 286 | Mean^a^ g/L. N = 287 | NR | NR | NR | NR | NR | NR |
|  | Teriflunomide (n = NR) | Week 84 | NR | Mean^a^ g/L. N = 260 | Mean^a^ g/L. N = 260 | NR | NR | NR | NR | NR | NR |
|  | Ofatumumab (n = NR) | Week 96 | NR | Mean and median (Mean = 10.487, Median = 10.33) ^a^ g/L. N = 172 | Mean and median (Mean = 0.812, Median = 0.71) ^a^ g/L. N = 172 | NR | NR | NR | NR | NR | NR |
|  | Teriflunomide (n = NR) | Week 96 | NR | Mean and median (Mean = 10.096, Median = 10.07) ^a^ g/L. N = 156 | Mean and median (Mean = 1.128, Median = 0.94) ^a^ g/L. N = 156 | NR | NR | NR | NR | NR | NR |
|  | Ofatumumab (n = NR) | Week 108 | NR | Mean^a^ g/L. N = 102 | Mean^a^ g/L. N = 102 | NR | NR | NR | NR | NR | NR |
|  | Teriflunomide (n = NR) | Week 108 | NR | Mean^a^ g/L. N = 91 | Mean^a^ g/L. N = 91 | NR | NR | NR | NR | NR | NR |
|  | Ofatumumab (n = NR) | Week 120 | NR | Mean (10.634) ^a^ g/L. N = 33 | Mean (0.887)^a^ g/L. N = 33 | NR | NR | NR | NR | NR | NR |
|  | Teriflunomide (n = NR) | Week 120 | NR | Mean (9.842)^a^ g/L. N = 26 | Mean (0.918)^a^ g/L. N = 26 | NR | NR | NR | NR | NR | NR |
|  | Ofatumumab (n = NR) | Week 120 | NR | Median (10.57). N = NR | Median (0.91). N = NR | NR | NR | NR | NR | NR | NR |
|  | Teriflunomide (n = NR) | Week 120 | NR | Median (10.01). N = NR | Median (0.84). N = NR | NR | NR | NR | NR | NR | NR |
| ASCLEPIOS II [2] | Ofatumumab (n = NR) | baseline | NR | Mean (10.082) ^a^ g/L. N = 480 | Mean (1.322)^a^ g/L. N = 480 | NR | NR | NR | NR | NR | NR |
|  | Teriflunomide (n = NR) | baseline | NR | Mean (10.118) ^a^ g/L. N = 473 | Mean (1.319)^a^ g/L. N = 473 | NR | NR | NR | NR | NR | NR |
|  | Ofatumumab (n = NR) | Week 4 | NR | Mean^a^ g/L. N = 475 | Mean^a^ g/L. N = 476 | NR | NR | NR | NR | NR | NR |
|  | Teriflunomide (n = NR) | Week 4 | NR | Mean^a^ g/L. N = 469 | Mean^a^ g/L. N = 469 | NR | NR | NR | NR | NR | NR |
|  | Ofatumumab (n = NR) | Week 12 | NR | Mean^a^ g/L. N = 467 | Mean^a^ g/L. N = 467 | NR | NR | NR | NR | NR | NR |
|  | Teriflunomide (n = NR) | Week 12 | NR | Mean^a^g/L. N = 463 | Mean^a^ g/L. N = 463 | NR | NR | NR | NR | NR | NR |
|  | Ofatumumab (n = NR) | Week 24 | NR | Mean^a^ g/L. N = 457 | Mean^a^ g/L. N = 457 | NR | NR | NR | NR | NR | NR |
|  | Teriflunomide (n = NR) | Week 24 | NR | Mean^a^ g/L. N = 451 | Mean^a^ g/L. N = 451 | NR | NR | NR | NR | NR | NR |
|  | Ofatumumab (n = NR) | Week 36 | NR | Mean^a^ g/L. N = 441 | Mean^a^ g/L. N = 441 | NR | NR | NR | NR | NR | NR |
|  | Teriflunomide (n = NR) | Week 36 | NR | Mean^a^ g/L. N = 433 | Mean^a^ g/L. N = 433 | NR | NR | NR | NR | NR | NR |
|  | Ofatumumab (n = NR) | Week 48 | NR | Mean (9.638)^a^ g/L. N = 425 | Mean (0.922)^a^ g/L. N = 425 | NR | NR | NR | NR | NR | NR |
|  | Teriflunomide (n = NR) | Week 48 | NR | Mean (8.913)^a^ g/L. N = 413 | Mean (1.1)^a^ g/L. N = 413 | NR | NR | NR | NR | NR | NR |
|  | Ofatumumab (n = NR) | Week 60 | NR | Mean^a^ g/L. N = 407 | Mean^a^ g/L. N = 408 | NR | NR | NR | NR | NR | NR |
|  | Teriflunomide (n = NR) | Week 60 | NR | Mean^a^ g/L. N = 402 | Mean^a^ g/L. N = 403 | NR | NR | NR | NR | NR | NR |
|  | Ofatumumab (n = NR) | Week 72 | NR | Mean^a^ g/L. N = 392 | Mean^a^ g/L. N = 392 | NR | NR | NR | NR | NR | NR |
|  | Teriflunomide (n = NR) | Week 72 | NR | Mean^a^ g/L. N = 387 | Mean^a^ g/L. N = 387 | NR | NR | NR | NR | NR | NR |
|  | Ofatumumab (n = NR) | Week 84 | NR | Mean^a^ g/L. N = 262 | Mean^a^g/L. N = 263 | NR | NR | NR | NR | NR | NR |
|  | Teriflunomide (n = NR) | Week 84 | NR | Mean^a^ g/L. N = 252 | Mean^a^ g/L. N = 252 | NR | NR | NR | NR | NR | NR |
|  | Ofatumumab (n = NR) | Week 96 | NR | Mean and median (Mean = 10.194, Median = 9.87) ^a^ g/L. N = 161 | Mean and median (Mean = 0.842, Median = 0.71) ^a^ g/L. N = 161 | NR | NR | NR | NR | NR | NR |
|  | Teriflunomide (n = NR) | Week 96 | NR | Mean and median (Mean = 9.803, Median = 9.51) ^a^ g/L. N = 160 | Mean and median (Mean = 1.014, Median = 0.93) ^a^ g/L. N = 160 | NR | NR | NR | NR | NR | NR |
|  | Ofatumumab (n = NR) | Week 108 | NR | Mean^a^ g/L. N = 92 | Mean^a^ g/L. N = 92 | NR | NR | NR | NR | NR | NR |
|  | Teriflunomide (n = NR) | Week 108 | NR | Mean^a^ g/L. N = 77 | Mean^a^ g/L. N = 77 | NR | NR | NR | NR | NR | NR |
|  | Ofatumumab (n = NR) | Week 120 | NR | Mean (10.175) ^a^ g/L. N = 23 | Mean (0.74) ^a^ g/L. N = 23 | NR | NR | NR | NR | NR | NR |
|  | Teriflunomide (n = NR) | Week 120 | NR | Mean (9.932)^a^ g/L. N = 18 | Mean (0.91)^a^ g/L. N = 18 | NR | NR | NR | NR | NR | NR |
|  | Ofatumumab (n = NR) | Week 120 | NR | Median (9.57). N = NR | Median (0.59). N = NR | NR | NR | NR | NR | NR | NR |
|  | Teriflunomide (n = NR) | Week 120 | NR | Median (9.65). N = NR | Median (0.92). N = NR | NR | NR | NR | NR | NR | NR |
| OBOE^b^  [7] | Ocrelizumab (n = 79) | baseline | NR | Median (0.79) IQR (0.63-1.28) CSF index. N = NR | Median (0.19) IQR (0.11-0.33) CSF index. N = NR | NR | NR | NR | NR | NR | NR |
|  | Ocrelizumab 600 mg + Week 12 lumbar (n = NR) | Week 12 | NR | NR | NR | NR | Median (IQR) = −4.1% (−15.1% to 6.3%) | Median (IQR) = 7% (−10.8% to 32.3%) | NR | NR | NR |
|  | Ocrelizumab 600 mg + Week 24 lumbar (n = NR) | Week 24 | NR | NR | NR | NR | Median (IQR) = −7% (−10.6% to 3.6%) | Median (IQR) = 1.4% (−4.7% to 21.2%) | NR | NR | NR |
|  | Ocrelizumab 600 mg + Week 52 lumbar (n = NR) | Week 52 | NR | NR | NR | NR | Median (IQR) = −9.5% (−20.4% to −0.1%) p< 0.02 | Median (IQR) = −9.2% (−22.2% to 12.1%) | NR | NR | NR |
| VELOCE^c^  [8, 9] | Ocrelizumab 600 mg (n = 68) | baseline | NR | Mean (10.25) SD (2.32) g/L. N = 68 | Mean (1.14) SD (0.66) g/L. N = 68 | NR | NR | NR | NR | NR | NR |
|  | Control (n = 34) | baseline | NR | Mean (10.63) SD (2.59) g/L. N = 34 | Mean (1.11) SD (0.51) g/L. N = 34 | NR | NR | NR | NR | NR | NR |
|  | Ocrelizumab 600 mg (n = 68) | Week 12 | NR | Mean (10.36) SD (2.38) g/L. N = 68 | Mean (0.99) SD (0.6) g/L. N = 68 | NR | NR | NR | NR | NR | NR |
|  | Control (n = 34) | Week 12 | NR | Mean (11.02) SD (2.78) g/L. N = 34 | Mean (1.14) SD (0.47) g/L. N = 34 | NR | NR | NR | NR | NR | NR |
|  | Ocrelizumab 600 mg (n = 68) | Week 24 | NR | Mean (10.21) SD (2.17) g/L. N = 67 | Mean (0.9) SD (0.61) g/L. N = 66 | NR | NR | NR | NR | NR | NR |
|  | Control (n = 34) | Week 24 | NR | NA | NR | NR | NR | NR | NR | NR | NR |
|  | Ocrelizumab 600 mg (n = 68) | Day 1 | Total Ig mean (SD) = 13.45 (2.75) g/L | | | NR | NR | NR | NR | NR | NR |
|  | Control (n = 34) | Day 1 | Total Ig mean (SD) = 14.05 (3.01) g/L | | | NR | NR | NR | NR | NR | NR |
|  | Ocrelizumab 600 mg (n = 68) | Day 85 | Total Ig mean (SD) = 13.45 (2.80) g/L | | | NR | NR | NR | NR | NR | NR |
|  | Control (n = 34) | Day 85 | Total Ig (SD) = 14.53 (3.19) g/L | | | NR | NR | NR | NR | NR | NR |
|  | Ocrelizumab 600 mg (n = 67) | Day 169 | Total Ig mean (SD) = 13.26 (2.62) g/L | | | NR | NR | NR | NR | NR | NR |
|  | Control (n = 0) | Day 169 | Total Ig mean (SD) = NR | | | NR | NR | NR | NR | NR | NR |
| OPERA I/II  [10-17] | Ocrelizumab | baseline (first dose of ocrelizumab) | Mean (2.13) g/l. N = NR | Mean (10.53) g/L. N = NR | Mean (1.35) g/L. N = NR | NR | NR | NR | n (%) < LLN = 17/1,444 (1.2), n (%) ≥ LLN = 1,427/1,444 (98.8) | n (%) < LLN = 7/1,446 (0.5), n (%) ≥ LLN = 1,439/1,446 (99.5) | n (%) < LLN = 7/1,446 (0.5), n (%) ≥ LLN = 1,439/1,446 (99.5) |
|  | Ocrelizumab | Week 24 | NR | NR | NR | NR | NR | NR | n (%) < LLN = 21/1,373 (1.5%), n (%) ≥ LLN = 1,352/1,373 (98.5) | n (%) < LLN = 10/1,381 (0.7), n (%) ≥ LLN = 1,371/1,381 (99.3) | n (%) < LLN = 90/1,371 (6.6), n (%) ≥ LLN = 1,281/1,371 (93.4) |
|  | Ocrelizumab | Week 48 | NR | NR | NR | NR | NR | NR | n (%) < LLN = 22/1,338 (1.6), n (%) ≥ LLN = 1316/1,338 (98.4) | n (%) < LLN = 10/1,344 (0.7), n (%) ≥ LLN = 1334/1,344 (99.3) | n (%) < LLN = 141/1,328 (10.6), n (%) ≥ LLN = 1187/1,328 (89.4) |
|  | Ocrelizumab | Week 72 | NR | NR | NR | NR | NR | NR | n (%) < LLN = 27/1,309 (2.1), n (%) ≥ LLN = 1282/1,309 (97.9) | n (%) < LLN = 19/1,313 (1.4), n (%) ≥ LLN = 1294/1,313 (98.6) | n (%) < LLN = 196/1,294 (15.1), n (%) ≥ LLN = 1098/1,294 (84.9) |
|  | Ocrelizumab | Week 96 | NR | NR | NR | NR | NR | NR | n (%) < LLN = 29/1,283 (2.3), n (%) ≥ LLN = 1254/1,283 (97.7) | n (%) < LLN = 17/1,285 (1.3), n (%) ≥ LLN = 1268/1,285 (98.7) | n (%) < LLN = 231/1,265 (18.3), n (%) ≥ LLN = 1034/1,265 (81.7) |
|  | Ocrelizumab | Week 120 | NR | NR | NR | NR | NR | NR | n (%) < LLN = 32/1,247 (2.6), n (%) ≥ LLN = 1215/1,247 (97.4) | n (%) < LLN = 30/1,249 (2.4), n (%) ≥ LLN = 1219/1,249 (97.6) | n (%) < LLN = 240/1,205 (19.9), n (%) ≥ LLN = 965/1,205 (80.1) |
|  | Ocrelizumab | Week 144 | NR | NR | NR | NR | NR | NR | n (%) < LLN = 32/1,207 (2.7), n (%) ≥ LLN = 1175/1,207 (97.3) | n (%) < LLN = 31/1,209 (2.6), n (%) ≥ LLN = 1178/1,209 (97.4) | n (%) < LLN = 267/1,158 (23.1), n (%) ≥ LLN = 891/1,158 (76.9) |
|  | Ocrelizumab | Week 168 | NR | NR | NR | NR | NR | NR | n (%) < LLN = 29/1,041 (2.8), n (%) ≥ LLN = 1012/1,041 (97.2) | n (%) < LLN = 26/1,043 (2.5), n (%) ≥ LLN = 1017/1,043 (97.5) | n (%) < LLN = 248/993 (25.0), n (%) ≥ LLN = 745/993 (75.0) |
|  | Ocrelizumab | Week 192 | NR | NR | NR | NR | NR | NR | n (%) < LLN = 29/811 (3.6), n (%) ≥ LLN = 782/811 (96.4) | n (%) < LLN = 29/812 (3.6), n (%) ≥ LLN = 783/812 (96.4) | n (%) < LLN = 195/759 (25.7), n (%) ≥ LLN = 564/759 (74.3) |
|  | Ocrelizumab | Week 216 | NR | NR | NR | NR | NR | NR | n (%) < LLN = 27/655 (4.1), n (%) ≥ LLN = 628/655 (95.9) | n (%) < LLN = 25/656 (3.8), n (%) ≥ LLN = 631/656 (96.2) | n (%) < LLN = 172/611 (28.2), n (%) ≥ LLN = 439/611 (71.8) |
|  | Ocrelizumab | Week 240 | NR | NR | NR | NR | NR | NR | n (%) < LLN = 31/606 (5.1), n (%) ≥ LLN = 575/606 (94.9) | n (%) < LLN = 33/607 (5.4), n (%) ≥ LLN = 574/607 (94.6) | n (%) < LLN = 164/556 (29.5), n (%) ≥ LLN = 392/556 (70.5) |
|  | Ocrelizumab | Week 264 | Mean (1.74) g/l. N = NR | Mean (8.79) g/L. N = NR | Mean (0.6) g/L. N = NR | Mean decrease = 21.3% | Mean decrease = 17.0% | Mean decrease = 58.1% | NR | NR | NR |
|  | Ocrelizumab, baseline IgG Q1 (lowest baseline levels) | 6 years | NR | NR | NR | NR | Mean change = −0.24 (−2.9%) g/L | NR | NR | NR | NR |
|  | Ocrelizumab, baseline IgG Q2 | 6 years | NR | NR | NR | NR | Mean change = − 0.32 (−3.2%) g/L | NR | NR | NR | NR |
|  | Ocrelizumab, baseline IgG Q3 | 6 years | NR | NR | NR | NR | Mean change = −0.40 (−3.6%) g/L | NR | NR | NR | NR |
|  | Ocrelizumab, baseline IgG Q4 (highest baseline levels) | 6 years | NR | NR | NR | NR | Mean change = −0.40 (−3.0%) g/L | NR | NR | NR | NR |
|  | Ocrelizumab 600 mg | baseline | Mean (2.11) 95% CI (digitized) (2.05-2.16) g/L. N = 822 | Mean (10.52) 95% CI (digitized) (10.4-10.7) g/L. N = 823 | Mean (1.34) 95% CI (digitized) (1.3-1.38) g/L. N = 823 | NR | NR | NR | <LLN = 1.5% | <LLN = 0.5% | <LLN = 0.1% |
|  | IFN β-1a for 96 weeks, switched to ocrelizumab 600 mg | baseline | Mean (2.12) 95% CI (unable to digitize) g/L. N = 823 | Mean (10.54) 95% CI (unable to digitize) g/L. N = 824 | Mean (1.37) 95% CI (digitized) (1.32-1.41) g/L. N = 824 | NR | NR | NR | < LLN = 1.2 | <LLN = 0.5% | <LLN = 1% |
|  | Ocrelizumab 600 mg | Week 24 | Digitized data: Mean (2.16) 95% CI (2.1-2.22) g/L. N = 777 | Mean (10.51) 95% CI (digitized) (10.4-10.7) g/L. N = 781 | Mean (1.06) 95% CI (digitized) (1.02-1.11) g/L. N = 776 | NR | NR | NR | NR | NR | NR |
|  | IFN β-1a for 96 weeks, switched to ocrelizumab 600 mg | Week 24 | Digitized data: Mean (2.31) 95% CI (2.25-2.38) g/L. N = 769 | Digitized data: Mean (11.3) 95% CI (11.1-11.4) g/L. N = 772 | Digitized data: Mean (1.45) 95% CI (1.39-1.49) g/L. N = 770 | NR | NR | NR | NR | NR | NR |
|  | Ocrelizumab 600 mg | Week 48 | Digitized data: Mean (2.13) 95% CI (2.08-2.2) g/L. N = 755 | Digitized data: Mean (10.4) 95% CI (10.3-10.6) g/L. N = 757 | Digitized data: Mean (0.961) 95% CI (0.924-1) g/L. N = 748 | NR | NR | NR | NR | NR | NR |
|  | IFN β-1a for 96 weeks, switched to ocrelizumab 600 mg | Week 48 | Digitized data: Mean (2.38) 95% CI (2.31-2.43) g/L. N = 710 | Digitized data: Mean (11.5) 95% CI (11.3-11.7) g/L. N = 711 | Digitized data: Mean (1.4) 95% CI (1.34-1.45) g/L. N = 711 | NR | NR | NR | NR | NR | NR |
|  | Ocrelizumab 600 mg | Week 72 | Digitized data: Mean (2.06) 95% CI (2.01-2.13) g/L. N = 739 | Digitized data: Mean (10.1) 95% CI (9.95-10.3) g/L. N = 741 | Digitized data: Mean (0.878) 95% CI (0.845-0.920) g/L. N = 729 | NR | NR | NR | NR | NR | NR |
|  | IFN β-1a for 96 weeks, switched to ocrelizumab 600 mg | Week 72 | Digitized data: Mean (2.4) 95% CI (2.33-2.46) g/L. N = 676 | Digitized data: Mean (11.5) 95% CI (11.3-11.7) g/L. N = 677 | Digitized data: Mean (1.34) 95% CI (1.29-1.39) g/L. N = 676 | NR | NR | NR | NR | NR | NR |
|  | Ocrelizumab 600 mg | Week 96 | Mean (2.06) 95% CI (digitized) (1.98-2.12) g/L. N = 718 | Mean (10.01) 95% CI (digitized) (9.88-10.2) g/L. N = 719 | Mean (0.84) 95% CI (digitized) (0.810-0.880) g/L. N = 703 | mean 3% decline | mean 5% decline | mean 40% decline | <LLN = 17/718 (2.4%), > 20% decline from baseline = 12% | <LLN = 11/719 (1.5%), > 20% decline from baseline = 10% | <LLN = 116/703 (16.5%) > 20% decline from baseline = 91% |
|  | IFN β-1a for 96 weeks, switched to ocrelizumab 600 mg | Week 96 | Mean (2.49) 95% CI (digitized) (2.41-2.55) g/L. N = 652 | Mean (11.6) 95% CI (digitized) (11.4-11.8) g/L. N = 653 | Mean (1.34) 95% CI (digitized) (1.29-1.39) g/L. N = 653 | NR | NR | NR | <LLN = 5/653 (0.8%), > 20% decline from baseline = 2% | < LLN = 2/653 (0.3%) | <LLN = 5/653 (0.8%), > 20% decline from baseline = 14% |
|  | Ocrelizumab 600 mg | Week 120 | Digitized data: Mean (1.99) 95% CI (1.93-2.05) g/L. N = 697 | Digitized data: Mean (9.68) 95% CI (9.52-9.82) g/L. N = 698 | Digitized data: Mean (0.775) 95% CI (0.747-0.807) g/L. N = 674 | NR | NR | NR | NR | NR | NR |
|  | IFN β-1a for 96 weeks, switched to ocrelizumab 600 mg | Week 120 | Digitized data: Mean (2.43) 95% CI (2.36-2.5) g/L. N = 601 | Digitized data: Mean (11.1) 95% CI (10.9-11.2) g/L. N = 602 | Digitized data: Mean (1.02) 95% CI (0.973-1.07) g/L. N = 600 | NR | NR | NR | NR | NR | NR |
|  | Ocrelizumab 600 mg | Week 144 | Digitized data: Mean (1.98) 95% CI (1.91-2.03) g/L. N = 663 | Digitized data: Mean (9.51) 95% CI (9.39-9.68) g/L. N = 664 | Digitized data: Mean (0.735) 95% CI (0.707-0.763) g/L. N = 639 | NR | NR | NR | NR | NR | NR |
|  | IFN β-1a for 96 weeks, switched to ocrelizumab 600 mg | Week 144 | Digitized data: Mean (2.34) 95% CI (2.28-2.42) g/L. N = 586 | Digitized data: Mean (10.7) 95% CI (10.5-10.9) g/L. N = 587 | Digitized data: Mean (0.898) 95% CI (0.861-0.940) g/L. N = 583 | NR | NR | NR | NR | NR | NR |
|  | Ocrelizumab 600 mg | Week 168 | Digitized data: Mean (1.92) 95% CI (1.86-1.98) g/L. N = 645 | Digitized data: Mean (9.5) 95% CI (9.35-9.67) g/L. N = 646 | Digitized data: Mean (0.702) 95% CI (0.675-0.740) g/L. N = 619 | NR | NR | NR | NR | NR | NR |
|  | IFN β-1a for 96 weeks, switched to ocrelizumab 600 mg | Week 168 | Digitized data: Mean (2.27) 95% CI (2.19-2.34) g/L. N = 574 | Digitized data: Mean (10.6) 95% CI (10.4-10.7) g/L. N = 575 | Digitized data: Mean (0.826) 95% CI (0.780-0.868) g/L. N = 569 | NR | NR | NR | NR | NR | NR |
|  | Ocrelizumab 600 mg | Week 192 | Digitized data: Mean (1.87) 95% CI (1.81-1.93) g/L. N = 627 | Digitized data: Mean (9.31) 95% CI (9.16-9.48) g/L. N = 628 | Digitized data: Mean (0.675) 95% CI (0.651-0.703) g/L. N = 584 | NR | NR | NR | <LLN = 3.7% | <LLN = 4.0% | <LLN = 25.2% |
|  | IFN β-1a for 96 weeks, switched to ocrelizumab 600 mg | Week 192 | Digitized data: Mean (2.19) 95% CI (2.12-2.26) g/L. N = 562 | Digitized data: Mean (10.3) 95% CI (10.1-10.5) g/L. N = 563 | Digitized data: Mean (0.77) 95% CI (0.731-0.808) g/L. N = 553 | NR | NR | NR | <LLN = 2.3% | <LLN = 1.1% | <LLN = 20.8% |
|  | Ocrelizumab 600 mg | Week 216 | Digitized data: Mean (1.84) 95% CI (1.78-1.9) g/L. N = 621 | Digitized data: Mean (9.18) 95% CI (9.02-9.33) g/L. N = 622 | Digitized data: Mean (0.646) 95% CI (0.618-0.674) g/L. N = 579 | NR | NR | NR | NR | NR | NR |
|  | IFN β-1a for 96 weeks, switched to ocrelizumab 600 mg | Week 216 | Digitized data: Mean (2.14) 95% CI (2.07-2.2) g/L. N = 550 | Digitized data: Mean (10.2) 95% CI (10-10.3) g/L. N = 551 | Digitized data: Mean (0.747) 95% CI (0.705-0.782) g/L. N = 531 | NR | NR | NR | NR | NR | NR |
|  | Ocrelizumab 600 mg | Week 240 | Digitized data: Mean (1.8) 95% CI (1.74-1.87) g/L. N = 606 | Digitized data: Mean (9.01) 95% CI (8.87-9.17) g/L. N = 607 | Digitized data: Mean (0.63) 95% CI (0.602-0.661) g/L. N = 556 | NR | NR | NR | NR | NR | NR |
|  | IFN β-1a for 96 weeks, switched to ocrelizumab 600 mg | Week 240 | Digitized data: Mean (2.1) 95% CI (2.02-2.17) g/L. N = 544 | Digitized data: Mean (10) 95% CI (9.87-10.2) g/L. N = 545 | Digitized data: Mean (0.724) 95% CI (0.684-0.766) g/L. N = 519 | NR | NR | NR | NR | NR | NR |
|  | Ocrelizumab 600 mg | Week 264 | Digitized data: Mean (1.75) 95% CI (1.68-1.81) g/L. N = 592 | Digitized data: Mean (8.85) 95% CI (8.71-9.05) g/L. N = 593 | Digitized data: Mean (0.614) 95% CI (0.588-0.644) g/L. N = 544 | NR | NR | NR | NR | NR | NR |
|  | IFN β-1a for 96 weeks, switched to ocrelizumab 600 mg | Week 264 | Digitized data: Mean (2.02) 95% CI (1.96-2.09) g/L. N = 536 | Digitized data: Mean (9.75) 95% CI (9.59-9.92) g/L. N = 537 | Digitized data: Mean (0.714) 95% CI (0.674-0.752) g/L. N = 506 | NR | NR | NR | NR | NR | NR |
|  | Ocrelizumab 600 mg | Week 288 | Mean (1.73) 95% CI (digitized) (1.66-1.79) NR. N = 580 | Mean (8.66) 95% CI (digitized) (8.54-8.86) g/L. N = 582 | Mean (0.62) 95% CI (digitized) (0.574-0.63) g/L. N = 530 | NR | Mean decrease = −0.32 g/L per year (−3.0% per year) - all ocrelizumab combined | Mean decrease = −0.78 g/L (mean relative reduction of 55.4%)- all ocrelizumab combined | <LLN = 6.9% | <LLN = 7% | <LLN = 31.5% |
|  | IFN β-1a for 96 weeks, switched to ocrelizumab 600 mg | Week 288 | Mean (1.97) 95% CI (digitized) (1.91-2.04) NR. N = 522 | Mean (9.55) 95% CI (digitized) (9.42-9.76) g/L. N = 523 | Mean (0.7) 95% CI (digitized) (0.656-0.731) g/L. N = 490 | NR | Mean decrease = −0.32 g/L per year (−3.0% per year) - all ocrelizumab combined | Mean decrease = −0.78 g/L (mean relative reduction of 55.4%)- all ocrelizumab combined | NR | NR | NR |
|  | Ocrelizumab 600 mg | Week 312 | Digitized data: Mean (1.71) 95% CI (1.64-1.77) g/L. N = 573 | Digitized data: Mean (8.67) 95% CI (8.51-8.82) g/L. N = 575 | Digitized data: Mean (0.593) 95% CI (0.568-0.62) g/L. N = 517 | NR | NR | NR | <LLN = 7.5% | <LLN = 7.7% | <LLN = 33.3% |
|  | IFN β-1a for 96 weeks, switched to ocrelizumab 600 mg | Week 312 | Digitized data: Mean (1.93) 95% CI (1.86-1.99) g/L. N = 512 | Digitized data: Mean (9.58) 95% CI (9.41-9.78) g/L. N = 513 | Digitized data: Mean (0.665) 95% CI (0.625-0.701) g/L. N = 479 | NR | NR | NR | <LLN = 3.9% | <LLN = 2.9% | <LLN = 28.2% |
|  | Ocrelizumab 600 mg (n = 562) | Week 336 | Mean (1.69) 95% CI (digitized) (1.61-1.74) g/L. N = 562 | Mean (8.5) 95% CI (digitized) (8.38-8.73) g/L. N = 566 | Mean (0.58) 95% CI (digitized) (0.546-0.599) g/L. N = 503 | NR | Serum IgG levels decreased at an average rate of −0.33 g/L per year (−2.99% per year) - all ocrelizumab | Mean reduction = -0.78 g/L (mean relative reduction of 55.8%) for all ocrelizumab | NR | NR | NR |
|  | IFN β-1a for 96 weeks, switched to ocrelizumab 600 mg (n = 506) | Week 336 | Mean (1.89) 95% CI (digitized) (1.8-1.94) g/L. N = 506 | Mean (9.41) 95% CI (digitized) (9.24-9.62) g/L. N = 508 | Mean (0.65) 95% CI (digitized) (0.61-0.686) g/L. N = 465 | NR | Serum IgG levels decreased at an average rate of −0.33 g/L per year (−2.99% per year) - all ocrelizumab | Mean reduction = -0.78 g/L (mean relative reduction of 55.8%) for all ocrelizumab | NR | NR | NR |
| OMS115102  [18] | Ofatumumab 100 mg to Week 24 then placebo (n = 8) | Week 24 | NR | NR | NR | Mean (SD) = 0.05 (0.16) g/L | Mean (SD) = -0.6 (1.0) g/L | Mean (SD) = -0.09 (0.14) g/L | NR | NR | NR |
|  | Ofatumumab 300 mg to Week 24 then placebo (n = 10) | Week 24 | NR | NR | NR | Mean (SD) = 0.08 (0.16) g/L | Mean (SD) = -0.1 (0.8) g/L | Mean (SD) = -0.13 (0.39) g/L | NR | NR | NR |
|  | Ofatumumab 700 mg to Week 24 then placebo (n = 6) | Week 24 | NR | NR | NR | Mean (SD) = 0.40 (0.40) g/L | Mean (SD) = 1.2 (1.0) g/L | Mean (SD) = -0.19 (0.09) g/L | NR | NR | NR |
|  | Placebo to Week 24 then ofatumumab 100 mg (n = 4) | Week 24 | NR | NR | NR | Mean (SD) = -0.07 (0.18) g/L | Mean (SD) = -0.5 (1.0) g/L | Mean (SD) = 0.25 (0.25) g/L | NR | NR | NR |
|  | Placebo to Week 24 then ofatumumab 300 mg (n = 4) | Week 24 | NR | NR | NR | Mean (SD) = 0 (0.44) g/L | Mean (SD) = -0.3 (0.9) g/L | Mean (SD) = -0.07 (0.19) g/L | NR | NR | NR |
|  | Placebo to Week 24 then ofatumumab 700 mg (n = 4) | Week 24 | NR | NR | NR | Mean (SD) = 0.49 (0.35) g/L | Mean (SD) = 1.4 (0.9) g/L | Mean (SD) = 0.04 (0.08) g/L | NR | NR | NR |
|  | Ofatumumab 100 mg to Week 24 then placebo (n = 8) | Week 48 | NR | NR | NR | Mean (SD) = 0.08 (0.24) g/L | Mean (SD) = -0.8 (1.2) g/L | Mean (SD) = -0.17 (0.24) g/L | NR | NR | NR |
|  | Ofatumumab 300 mg to Week 24 then placebo (n = 11) | Week 48 | NR | NR | NR | Mean (SD) = -0.03 (0.39) g/L | Mean (SD) = 0.5 (1.6) g/L | Mean (SD) = -0.21 (0.22) g/L | NR | NR | NR |
|  | Ofatumumab 700 mg to Week 24 then placebo (n = 7) | Week 48 | NR | NR | NR | Mean (SD) = 0.13 (0.11) g/L | Mean (SD) = 1.0 (0.7) g/L | Mean (SD) = -0.18 (0.24) g/L | NR | NR | NR |
|  | Placebo to Week 24 then ofatumumab 100 mg (n = 4) | Week 48 | NR | NR | NR | Mean (SD) = -0.00 (0.24) g/L | Mean (SD) = -0.6 (1.2) g/L | Mean (SD) = -0.24 (0.24) g/L | NR | NR | NR |
|  | Placebo to Week 24 then ofatumumab 300 mg (n = 3) | Week 48 | NR | NR | NR | Mean (SD) = 0.05 (0.21) g/L | Mean (SD) = 0.2 (1.6) g/L | Mean (SD) = -0.40 (0.17) g/L | NR | NR | NR |
|  | Placebo to Week 24 then ofatumumab 700 mg (n = 4) | Week 48 | NR | NR | NR | Mean (SD) = 0.29 (0.26) g/L | Mean (SD) = 1.2 (0.6) g/L | Mean (SD) = -0.13 (0.04) g/L | NR | NR | NR |
|  | Ofatumumab 100 mg to Week 24 then placebo (n = 1) | Week 104 | NR | NR | NR | Mean (SD) = -0.23 (NA) g/L | Mean (SD) = -0.6 (NA) g/L | Mean (SD) = -0.07 (NA) g/L | NR | NR | NR |
| NCT00676715  [19] | 4 cycles of ocrelizumab (n = 49) | Week 120 | NR | NR | NR | NR | Reduced by 6.94 ± 11.94% | Reduced by 39.54 ± 15.38% | NR | NR | NR |
|  | 3 cycles of ocrelizumab (n = 46) (placebo-ocrelizumab group) | Week 120 | NR | NR | NR | NR | NR | Reduced by 34.87 ± 22.56% | NR | NR | NR |
| ASCLEPIOS I/II, APLIOS, APOLITOS, ALITHIOS  [20] | Ofatumumab | Week 0 | NR | Mean (10.31) SE (unable to digitize) g/L. N = 1,269 | Mean (1.34) SE (digitized) (0.05) g/L. N = 1,271 | NR | NR | NR | NR | NR | NR |
|  | Teriflunomide | Week 0 | NR | Mean (10.34) SE (unable to digitize) g/L. N = 675 | Mean (1.36) SE (digitized) (0.04) g/L. N = 675 | NR | NR | NR | NR | NR | NR |
|  | Ofatumumab | Week 4 | NR | Digitized data: Mean (10.1) SE (0.1) g/L. N = 1,233 | Digitized data: Mean (1.25) SE (0.01) g/L. N = 1,235 | NR | NR | NR | NR | NR | NR |
|  | Teriflunomide | Week 4 | NR | Digitized data: Mean (9.77) SE (0.09) g/L. N = 672 | Digitized data: Mean (1.3) SE (0.03) g/L. N = 672 | NR | NR | NR | NR | NR | NR |
|  | Ofatumumab | Week 12 | NR | Digitized data: Mean (10.2) SE (0.1) g/L. N = 1,248 | Digitized data: Mean (1.13) SE (0.02) g/L. N = 1,250 | NR | NR | NR | NR | NR | NR |
|  | Teriflunomide | Week 12 | NR | Digitized data: Mean (9.29) SE (0.1) g/L. N = 673 | Digitized data: Mean (1.17) SE (0.03) g/L. N = 673 | NR | NR | NR | NR | NR | NR |
|  | Ofatumumab | Week 24 | NR | Digitized data: Mean (10.1) SE (0) g/L. N = 1,219 | Digitized data: Mean (1.04) SE (0.02) g/L. N = 1,221 | NR | NR | NR | NR | NR | NR |
|  | Teriflunomide | Week 24 | NR | Digitized data: Mean (9.07) SE (0.1) g/L. N = 666 | Digitized data: Mean (1.09) SE (0.03) g/L. N = 666 | NR | NR | NR | NR | NR | NR |
|  | Ofatumumab | Week 36 | NR | Digitized data: Mean (9.87) SE (0.09) g/L. N = 1,180 | Digitized data: Mean (0.968) SE (0.017) g/L. N = 1,182 | NR | NR | NR | NR | NR | NR |
|  | Teriflunomide | Week 36 | NR | Digitized data: Mean (8.91) SE (0.08) g/L. N = 672 | Digitized data: Mean (1.08) SE (0.03) g/L. N = 672 | NR | NR | NR | NR | NR | NR |
|  | Ofatumumab | Week 48 | NR | Digitized data: Mean (9.94) SE (0.06) g/L. N = 1,132 | Digitized data: Mean (0.927) SE (0.02) g/L. N = 1,134 | NR | NR | absolute mean, 0.93 g/L; % change, −31.8% | NR | NR | NR |
|  | Teriflunomide | Week 48 | NR | Digitized data: Mean (9.09) SE (0.1) g/L. N = 669 | Digitized data: Mean (1.1) SE (0.03) g/L. N = 669 | NR | NR | NR | NR | NR | NR |
|  | Ofatumumab | Week 60 | NR | Digitized data: Mean (10.1) SE (0.1) g/L. N = 1,083 | Digitized data: Mean (0.895) SE (0.022) g/L. N = 1,088 | NR | NR | NR | NR | NR | NR |
|  | Teriflunomide | Week 60 | NR | Digitized data: Mean (9.37) SE (0.11) g/L. N = 666 | Digitized data: Mean (1.08) SE (0.03) g/L. N = 666 | NR | NR | NR | NR | NR | NR |
|  | Ofatumumab | Week 72 | NR | Digitized data: Mean (10.3) SE (0.1) g/L. N = 902 | Digitized data: Mean (0.863) SE (0.02) g/L. N = 906 | NR | NR | NR | NR | NR | NR |
|  | Teriflunomide | Week 72 | NR | Digitized data: Mean (9.63) SE (0.11) g/L. N = 670 (6 switched) | Digitized data: Mean (1.09) SE (0.03) g/L. N = 670 (6 switched) | NR | NR | NR | NR | NR | NR |
|  | Ofatumumab | Week 84 | NR | Digitized data: Mean (10.3) SE (0.1) g/L. N = 961 | Digitized data: Mean (0.839) SE (0.021) g/L. N = 965 | NR | NR | NR | NR | NR | NR |
|  | Teriflunomide - switch to OFA | Week 84 | NR | Digitized data: Mean (9.96) SE (0.14) g/L. N = 635 (158 switched) | Digitized data: Mean (1.08) SE (0.03) g/L. N = 635 (158 switched) | NR | NR | NR | NR | NR | NR |
|  | Ofatumumab | Week 96 | NR | Digitized data: Mean (10.3) SE (0.1) g/L. N = 758 | Digitized data: Mean (0.815) SE (0.019) g/L. N = 768 | NR | NR | NR | NR | NR | NR |
|  | Teriflunomide - switch to OFA | Week 96 | NR | Digitized data: Mean (10.2) SE (0.1) g/L. N = 634 (321 switched) | Digitized data: Mean (1.07) SE (0.03) g/L. N = 634 (321 switched) | NR | NR | NR | NR | NR | NR |
|  | Ofatumumab | Week 108 | NR | Digitized data: Mean (10.2) SE (0.1) g/L. N = 712 | Digitized data: Mean (0.79) SE (0.017) g/L. N = 715 | NR | NR | NR | NR | NR | NR |
|  | Teriflunomide - switch to OFA | Week 108 | NR | Digitized data: Mean (10.4) SE (0.1) g/L. N = 635 (468 switched) | Digitized data: Mean (1.03) SE (0.03) g/L. N = 635 (468 switched) | NR | NR | NR | NR | NR | NR |
|  | Ofatumumab | Week 120 | NR | Digitized data: Mean (10.3) SE (0) g/L. N = 602 | Digitized data: Mean (0.783) SE (0.025) g/L. N = 603 | NR | NR | NR | NR | NR | NR |
|  | Teriflunomide - switch to OFA | Week 120 | NR | Digitized data: Mean (10.4) SE (0.2) g/L. N = 567 (495 switched) | Digitized data: Mean (0.951) SE (0.03) g/L. N = 567 (495 switched) | NR | NR | NR | NR | NR | NR |
|  | Ofatumumab | Week 132 | NR | Digitized data: Mean (10.2) SE (0.1) g/L. N = 514 | Digitized data: Mean (0.75) SE (0.022) g/L. N = 515 | NR | NR | NR | NR | NR | NR |
|  | Teriflunomide - switch to OFA, long-term OFA | Week 132 | NR | Digitized data: Mean (10.5) SE (0.1) g/L. N = 499 (all switched) | Digitized data: Mean (0.899) SE (0.023) g/L. N = 506 (all switched) | NR | NR | NR | NR | NR | NR |
|  | Ofatumumab | Week 144 | NR | Digitized data: Mean (10.3) SE (0.1) g/L. N = 469 | Digitized data: Mean (0.724) SE (0.023) g/L. N = 477 | NR | NR | NR | NR | NR | NR |
|  | Teriflunomide - switch to OFA, long-term OFA | Week 144 | NR | Digitized data: Mean (10.6) SE (0.1) g/L. N = 447 (all switched) | Digitized data: Mean (0.855) SE (0.033) g/L. N = 451 (all switched) | NR | NR | NR | NR | NR | NR |
|  | Ofatumumab | Week 156 | NR | Digitized data: Mean (10.2) SE (0.1) g/L. N = 345 | Digitized data: Mean (0.689) SE (0.019) g/L. N = 350 | NR | NR | NR | NR | NR | NR |
|  | Teriflunomide - switch to OFA, long-term OFA | Week 156 | NR | Digitized data: Mean (10.7) SE (0.1) g/L. N = 350 (all switched) | Digitized data: Mean (0.827) SE (0.038) g/L. N = 359 (all switched) | NR | NR | NR | NR | NR | NR |
|  | Ofatumumab | Week 168 | NR | Mean (10.25) SE (digitized) (0.05) g/L. N = 241 | Mean (0.71) SE (digitized) (0.031) g/L. N = 248 | NR | NR | absolute mean, 0.71 g/L; % change, −46% | NR | NR | NR |
|  | Teriflunomide - switch to OFA, long-term OFA | Week 168 | NR | Mean (10.48) SE (digitized) (0.12) g/L. N = 215 (all switched) | Mean (0.75) SE (digitized) (0.047) g/L. N = 220 (all switched) | NR | NR | NR | NR | NR | NR |
|  | Ofatumumab | Any time postbaseline | NR | NR | NR | NR | 1.10% | -46% | NR | <LLN = 1.5% (30/1969) | <LLN = 23.1% (454/1969) |

CI = confidence interval; CSF = cerebrospinal fluid; g/L = gram per liter; Ig = immunoglobulin; IFN = interferon; IQR = interquartile range; LLN = lower limit of normal; N = number; NR = not reported; OFA = ofatumumab; SE = standard error; SD = standard deviation.

Note: When data were not directly reported, but were available in a figure, this data was digitized. Where data have been digitized, this is noted within the results within this table.

^a^ Mean and/or SE Reported in references 437/IS9/IS32 figures but not clear enough to be digitized.

^b^ This study only reported CSF Ig data.

^c^ This study also reports vaccine specific geometric mean antibody titers and response for tetanus, pneumococcal, influenza, KLH neoantigen.

Table S6. Outcomes Reported From Real-World Studies

| Author (year), country | Treatment & timepoint | Mean or median | | | Change from baseline | | |
| --- | --- | --- | --- | --- | --- | --- | --- |
|  |  | IgA | IgG | IgM | IgA | IgG | IgM |
| Prezioso et al. [21], Italy | Ocrelizumab (n = 42) Baseline | NR | Mean (960.08) SD (202.22) mg/dL | Mean (127.95) SD (53.5) mg/dL | NR | NR | NR |
|  | Ocrelizumab (n = 42)  6 months |  | Mean (964.22) SD (180.05) mg/dL | Mean (98.19) SD (55.57) mg/dL |  |  |  |
|  | Ocrelizumab (n = 42)  12 months |  | Mean (978.14) SD (191.09) mg/dL | Mean (75.04) SD (41.93) mg/dL |  |  |  |
| van Lierop et al. [22], Netherlands | Ocrelizumab direct switch (n = 27)  Screening | NR | Median (9.8) IQR (8.2-10.6) g/L | NR | NR | NR | NR |
|  | Ocrelizumab indirect switch (n = 15)  Screening |  | Median (9.1) IQR (7.9-10.1) g/L |  |  |  |  |
|  | Ocrelizumab direct switch (n = 27)  FU1 - second dose (12 weeks) |  | Median (9.45) IQR (7.6-10.5) g/L |  |  |  |  |
|  | Ocrelizumab indirect switch (n = 15)  FU1 - second dose (2 weeks) |  | Median (8.7) IQR (7.8-10.6) g/L |  |  |  |  |
|  | Ocrelizumab direct switch (n = 27)  FU2 - first 600 mg dose (30 weeks) |  | Median (9.15) IQR (7.9-10.2) g/L |  |  |  |  |
|  | Ocrelizumab indirect switch (n = 15)  FU2 - first 600 mg dose (24 weeks) |  | Median (9.6) IQR (8.1-10.9) g/L |  |  |  |  |
|  | Ocrelizumab direct switch (n = 27)  FU3 - second 600 mg dose (54 weeks) |  | Median (9.6) IQR (7.9-10.1) g/L |  |  |  |  |
|  | Ocrelizumab indirect switch (n = 15)  FU3 - second 600 mg dose (48 weeks) |  | Median (9.7) IQR (8.1-11.0) g/L |  |  |  |  |
|  | Ocrelizumab direct switch (n = 27)  FU4 |  | Median (9.3) IQR (7.8-10.1) g/L |  |  |  |  |
|  | Ocrelizumab indirect switch (n = 15)  FU4 |  | Median (9.2) IQR (8.0-11.0) g/L |  |  |  |  |
|  | Ocrelizumab direct switch (n = 27)  FU5 |  | Median (9.4) IQR (7.1-10.8) g/L |  |  |  |  |
|  | Ocrelizumab indirect switch (n = 15)  FU5 |  | Median (9.6) IQR (8.0-10.7) g/L |  |  |  |  |
| Edgar et al. [23]^a^, US [abstract and presentation] | Ocrelizumab SR (n = 13)  Baseline | NR | Median (1,012) IQR digitized (810-1290) mg/dL | Median (66) IQR digitized (31.4-125) mg/dL | NR | NR | NR |
|  | Ocrelizumab RP (n = 114)  Baseline |  | Median (873.5) IQR digitized (670-982) mg/dL | Median (76) IQR digitized (45.3-108) mg/dL |  |  |  |
|  | Ocrelizumab SR (n = 14)  Course 1 |  | Median (1,031) IQR digitized (819-1320) mg/dL | Median (56) IQR digitized (25.5-119) mg/dL |  |  |  |
|  | Ocrelizumab RP (n = 112)  Course 1 |  | Median (841.5) IQR digitized (665-964) mg/dL | Median (59) IQR digitized (39-100) mg/dL |  |  |  |
|  | Ocrelizumab SR (n = 13)  Course 2 |  | Median (948) IQR digitized (795-1220) mg/dL | Median (45) IQR digitized (24.9-71.4) mg/dL |  |  |  |
|  | Ocrelizumab RP (n = 100)  Course 2 |  | Median (777.5) IQR digitized (626-955) mg/dL | Median (47.5) IQR digitized (34-77) mg/dL |  |  |  |
|  | Ocrelizumab SR (n = 7)  Course 3 |  | NR | Median (39) IQR digitized (21.2-82) mg/dL |  |  |  |
|  | Ocrelizumab RP (n = 49)  Course 3 |  | NR | Median (38) IQR digitized (28.5-67) mg/dL |  |  |  |
| Evertsson et al. [24] ^a^, US | Ocrelizumab (n = 161)  Baseline | NR | Digitized: Median (8.87) IQR (7.24-10.2) g/L | Digitized: Median (0.656) IQR (0.433-1.08) g/L | NR | NR | NR |
|  | Ocrelizumab (n = 161)  6 months |  | Digitized: Median (8.57) IQR (7.22-9.9) g/L | Digitized: Median (0.661) IQR (0.432-1.02) g/L |  | Mean decrease of 0.16 g/L (CI, 0.01-0.31, *P* = 0.039) with each infusion according to MEM analysis. GEE analysis results in a reduction of 0.001 g/L per day and 0.18 g/L per infusion (*P* = 0.102) | Mean decrease of 0.11 g/L (CI, 0.09-0.14) with each infusion with MEM analysis. GEE analysis results in decrease of 0.18 g/L, *P* < 0.001 |
|  | Ocrelizumab (n = 161)  12 months |  | Digitized: Median (8.52) IQR (7.1-9.38) g/L | Digitized: Median (0.634) IQR (0.352-0.909) g/L |  | Mean decrease of 0.16 g/L (CI, 0.01-0.31, *P* = 0.039) with each infusion according to MEM analysis. GEE analysis results in a reduction of 0.001 g/L per day and 0.18 g/L per infusion (*P* = 0.102) | Mean decrease of 0.11 g/L (CI, 0.09-0.14) with each infusion with MEM analysis. GEE analysis results in decrease of 0.18 g/L, *P* < 0.001 |
|  | Ocrelizumab < 40 years (n = NR)  Baseline |  | Mean (8.82) SD (2) g/L | NR |  | NR | NR |
|  | Ocrelizumab < 40 years (n = NR)  12 months |  | Mean (8.55) SD (1.4) g/L | NR |  | NR | NR |
|  | Ocrelizumab 40-50 years (n = NR)  Baseline |  | Mean (8.79) SD (2.2) g/L | NR |  | NR | NR |
|  | Ocrelizumab 50-40 years (n = NR)  12 months |  | Mean (8.75) SD (2.8) g/L | NR |  | NR | NR |
|  | Ocrelizumab > 50 years (n = NR)  Baseline |  | Mean (8.76) SD (2.2) g/L | NR |  | NR | NR |
|  | Ocrelizumab > 50 years (n = NR)  12 months |  | Mean (7.99) SD (2) g/L | NR |  | NR | NR |
| Evertsson et al. [25] ^a^, US [abstract and poster] | Ocrelizumab (n = 161)  Up to 12 months | NR | NR | NR | NR | Reduction of 0.17 g/L (CI, 0.04-0.31) with each infusion | Reduction of 0.12 g/L (CI, 0.10-0.14) with each infusion according to poster and 11 mg/dL (CI, 9-14) according to abstract |
|  | Ocrelizumab (n = 161) 6 months |  | Digitized: Median (8.9) IQR (7.16-10.3) g/L | Digitized: Median (0.667) IQR (0.405-1.09) g/L |  | NR | NR |
|  | Ocrelizumab (n = 161) 12 months |  | Digitized: Median (8.56) IQR (7.06-9.8) g/L | Digitized: Median (0.582) IQR (0.359-0.980) g/L |  | NR | NR |
|  | Ocrelizumab (n = 161) Baseline |  | Digitized: Median (8.36) IQR (6.39-9.45) g/L | Digitized: Median (0.497) IQR (0.314-0.850) g/L |  | NR | NR |
| Lopez Ruiz et al. [26], Spain [abstract and presentation] | Ocrelizumab (n = 31) Baseline | NR | Mean (963.5) SD (167.4) mg/dL | Mean (99.9) SD (49.8) mg/dL | NR | NR | NR |
|  | Ocrelizumab (n = 29) 6 months |  | Mean (997.6) SD (192.2) mg/dL | Mean (89.6) SD (47.7) mg/dL |  |  |  |
|  | Ocrelizumab (n = 27) 12 months |  | Mean (999.9) SD (222.8) mg/dL | Mean (73.9) SD (41.03) mg/dL |  |  |  |
|  | Ocrelizumab (n = NR) 18 months |  | Mean (869.9) SD (186.3) mg/dL | Mean (69.3) SD (42.6) mg/dL |  |  |  |

CI = confidence interval; GEE = generalized estimating equation; Ig = immunoglobulin; IQR = interquartile range; MEM = maximum entropy method; NR = not reported; RP = remaining population; RWE = real-world evidence; SD = standard deviation; SR = super response; US = United States.

^a^ Data for Rituximab (RTX) was available but not extracted.

## Appendix References

[1] Wiendl H, De Seze J, Bar-Or A, Correale J, Cross AH, Kappos L, et al. Serum immunoglobulin levels and infection risk in the phase 3 trials of ofatumumab in relapsing multiple sclerosis. Mult Scler J. 2020;26(3 Suppl):233-4.

[2] de Seze J, Bar-Or A, Correale J, Cross AH, Kappos L, Selmaj K, et al. Effect of ofatumumab on serum immunoglobulin levels and infection risk in relapsing multiple sclerosis patients from the phase 3 ASCLEPIOS I and II trials. Eur J Neurol. 2020;27:1295-6.

[3] Hauser SL, Bar-Or A, Cohen JA, Comi G, Correale J, Coyle PK, et al. Ofatumumab versus teriflunomide in multiple sclerosis. N Engl J Med. 2020;383(6):546-57.

[4] de Seze J, Bar-Or A, Correale J, Cross AH, Kappos L, Selmaj K, et al. Effect of ofatumumab on serum immunoglobulin levels and infection risk in relapsing multiple sclerosis patients from the Phase 3 ASCLEPIOS I and II trials. Int J MS Care. 2020;22(s2).

[5] Bar-Or A, De Seze J, Correale J, Cross A, Kappos L, Selmaj K, et al. Effect of ofatumumab on serum immunoglobulin levels and infection risk in relapsing multiple sclerosis (RMS) patients from the phase 3 ASCLEPIOS I and II trials (1300). Neurology. 2020;96(15 Supplement).

[6] European Medicines Agency. Kesimpta summary of product characteristics. 24 June 2021. Available at: <https://www.ema.europa.eu/en/documents/product-information/kesimpta-epar-product-information_en.pdf>. Accessed: 10 September 2021.

[7] Weber M, Von Büdingen HC, Bar-Or A, Herman A, Harp C, Pei J, et al. Modulation of cerebrospinal fluid immunoglobulins by ocrelizumab treatment. Mult Scler J. 2020;26(3 Suppl):171-2.

[8] Bar-Or A, Calkwood JC, Chognot C, Evershed J, Fox EJ, Herman A, et al. Effect of ocrelizumab on vaccine responses in patients with multiple sclerosis: the VELOCE study. Neurology. 2020;95(14):e1999-e2008.

[9] Hoffmann-La Roche. A study to evaluate the effects of ocrelizumab on immune responses in participants with relapsing forms of multiple sclerosis. ClinicalTrials.gov identifier: NCT02545868. Updated 28 April 2022. Available at: <https://www.cochranelibrary.com/central/doi/10.1002/central/CN-01492132/full>.

[10] Hauser SL, Kappos L, Arnold DL, Bar-Or A, Brochet B, Naismith RT, et al. Five years of ocrelizumab in relapsing multiple sclerosis: OPERA studies open-label extension. Neurology. 2020;95(13):e1854-e67.

[11] Derfuss T, Weber M, Hughes R, Eggebrecht J, Wang Q, Sauter A, et al. Serum immunoglobulin levels and risk of serious infections in the pivotal phase III trials of ocrelizumab in multiple sclerosis and their open-label extensions. Clin Neurophysiol. 2020;131(4):e196.

[12] Derfuss T, Weber MS, Hughes R, Wang Q, Sauter A, Koendgen H, et al. Serum immunoglobulin levels and risk of serious infections in the pivotal Phase III trials of ocrelizumab in multiple sclerosis and their open-label extensions. Mult Scler J. 2019;25:20-1.

[13] Bar-Or A, Bermel R, Weber MS, Hughes R, Lin CJ, Wang J, et al. Serum IG levels and risk of serious infections by baseline IG quartile in the pivotal phase III trials and open-label extensions of ocrelizumab in multiple sclerosis. Neurology. 2020;94(15).

[14] Hauser SL, Kappos L, Montalban X, Craveiro L, Chognot C, Hughes R, et al. Safety of ocrelizumab in patients with relapsing and primary progressive multiple sclerosis. Neurology. 2021 Sep 2.

[15] US Food and Drug Administration. Ocrevus medical review(s). 5 November 2015. Available at: <https://www.accessdata.fda.gov/drugsatfda_docs/nda/2017/761053Orig1s000MedR.pdf>. Accessed: 10 September 2021.

[16] European Medicines Agency. Ocrevus assessment report. Procedure No. EMEA/H/C/004043/0000. 9 November 2017. Available at: <https://www.ema.europa.eu/en/documents/assessment-report/ocrevus-epar-public-assessment-report_en.pdf>. Accessed: 10 September 2021.

[17] Hauser SL, Bar-Or A, Comi G, Giovannoni G, Hartung HP, Hemmer B, et al. Ocrelizumab versus interferon beta-1a in relapsing multiple sclerosis. N Engl J Med. 2017;376(3):221-34.

[18] GlaxoSmithKline. Ofatumumab dose-finding in relapsing remitting multiple sclerosis (RRMS) patients. ClinicalTrials.gov identifier: NCT00640328. Updated 11 April 2017. Available at: <https://ClinicalTrials.gov/show/NCT00640328>.

[19] Baker D, Pryce G, James LK, Marta M, Schmierer K. The ocrelizumab phase II extension trial suggests the potential to improve the risk: benefit balance in multiple sclerosis. Mult Scler Relat Disord. 2020;44:102279.

[20] Wiendl H, de Seze J, Bar-Or A, Correale J, Cross AH, Kappos L, et al. Effect of ofatumumab on serum immunoglobulin levels and infection risk in patients with relapsing multiple sclerosis over 3.5 years. 37th Congress of the European Committee for Treatment and Research in Multiple Sclerosis. Digital Experience; 2021.

[21] Prezioso C, Grimaldi A, Landi D, Nicoletti CG, Brazzini G, Piacentini F, et al. Risk assessment of progressive multifocal leukoencephalopathy in multiple sclerosis patients during 1 year of ocrelizumab treatment. Viruses. 2021;13(9):1684.

[22] van Lierop ZYGJ, Toorop AA, Coerver EME, Willemse EAJ, Strijbis EMM, Kalkers NF, et al. Ocrelizumab after natalizumab in JC-virus positive relapsing remitting multiple sclerosis patients. Mult Scler J Exp Transl Clin. 2021;7(2):20552173211013831.

[23] Edgar N, Hoyt T, Foley J. Can we predict ocrelizumab super responder status in relapsing remitting multiple sclerosis patients? Mult Scler J. 2020;26(3 Suppl):267.

[24] Evertsson B, Hoyt T, Christensen A, Nimer FAL, Foley J, Piehl F. A comparative study of tolerability and effects on immunoglobulin levels and CD19 cell counts with ocrelizumab vs low dose of rituximab in multiple sclerosis. Mult Scler J Exp Transl Clin. 2020;6(4):2055217320964505.

[25] Evertsson B, Hoyt T, Christensen A, Al Nimer F, Foley J, Piehl F. Comparative study of tolerability and effects on immunoglobulin levels and CD19 cell counts with ocrelizumab vs rituximab in multiple sclerosis. Mult Scler J. 2019;25:515-6.

[26] Lopez Ruiz R, Eichau S, Guerra Hiraldo J, Dotor Garcia-Soto J, Ruiz de Arcos M, Ruiz-Pena J. Real world data on the use of ocrelizumab. Incidence of lymphopenia, B-cell and immunoglobulins evolution. 37th Congress of the European Committee for Treatment and Research in Multiple Sclerosis. Digital Experience; 2021.
